# A toolbox for class I HDACs reveals isoform specific roles in gene regulation and protein acetylation

**DOI:** 10.1371/journal.pgen.1010376

**Published:** 2022-08-22

**Authors:** Lena Hess, Verena Moos, Arnel A. Lauber, Wolfgang Reiter, Michael Schuster, Natascha Hartl, Daniel Lackner, Thorina Boenke, Anna Koren, Paloma M. Guzzardo, Brigitte Gundacker, Anna Riegler, Petra Vician, Claudia Miccolo, Susanna Leiter, Mahesh B. Chandrasekharan, Terezia Vcelkova, Andrea Tanzer, Jun Qi Jun, James Bradner, Gerald Brosch, Markus Hartl, Christoph Bock, Tilmann Bürckstümmer, Stefan Kubicek, Susanna Chiocca, Srividya Bhaskara, Christian Seiser

**Affiliations:** 1 Center for Anatomy and Cell Biology, Medical University of Vienna, Vienna, Austria; 2 Mass Spectrometry Core Facility, Max Perutz Labs, Vienna BioCenter, Vienna, Austria; 3 Department of Biochemistry and Cell Biology, Max Perutz Labs, University of Vienna, Vienna BioCenter, Vienna, Austria; 4 CeMM Research Center for Molecular Medicine of the Austrian Academy of Sciences, Vienna, Austria; 5 Horizon Genomics, Vienna, Austria; 6 Department of Experimental Oncology, IEO, European Institute of Oncology IRCCS, Milan, Italy; 7 Department of Radiation Oncology and Huntsman Cancer Institute, University of Utah School of Medicine, Salt Lake City, Utah, United States of America; 8 Dana-Farber Cancer Institute, Boston, Massachusetts, United States of America; 9 Institute of Molecular Biology, Innsbruck Medical University, Innsbruck, Austria; 10 Institute of Artificial Intelligence, Center for Medical Statistics, Informatics, and Intelligent Systems, Medical University of Vienna, Vienna, Austria; Friedrich Miescher Institute for Biomedical Research, SWITZERLAND

## Abstract

The class I histone deacetylases are essential regulators of cell fate decisions in health and disease. While pan- and class-specific HDAC inhibitors are available, these drugs do not allow a comprehensive understanding of individual HDAC function, or the therapeutic potential of isoform-specific targeting. To systematically compare the impact of individual catalytic functions of HDAC1, HDAC2 and HDAC3, we generated human HAP1 cell lines expressing catalytically inactive HDAC enzymes. Using this genetic toolbox we compare the effect of individual HDAC inhibition with the effects of class I specific inhibitors on cell viability, protein acetylation and gene expression. Individual inactivation of HDAC1 or HDAC2 has only mild effects on cell viability, while HDAC3 inactivation or loss results in DNA damage and apoptosis. Inactivation of HDAC1/HDAC2 led to increased acetylation of components of the COREST co-repressor complex, reduced deacetylase activity associated with this complex and derepression of neuronal genes. HDAC3 controls the acetylation of nuclear hormone receptor associated proteins and the expression of nuclear hormone receptor regulated genes. Acetylation of specific histone acetyltransferases and HDACs is sensitive to inactivation of HDAC1/HDAC2. Over a wide range of assays, we determined that in particular HDAC1 or HDAC2 catalytic inactivation mimics class I specific HDAC inhibitors. Importantly, we further demonstrate that catalytic inactivation of HDAC1 or HDAC2 sensitizes cells to specific cancer drugs. In summary, our systematic study revealed isoform-specific roles of HDAC1/2/3 catalytic functions. We suggest that targeted genetic inactivation of particular isoforms effectively mimics pharmacological HDAC inhibition allowing the identification of relevant HDACs as targets for therapeutic intervention.

## Author summary

Histone deacetylases (HDACs) remove acetyl groups from histones and non-histone proteins. Dysregulated acetylation has been attributed to various disease states, including cancer, immunological and neurological diseases. Due to the potential reversibility of epigenetic or post-translational modifications, the pharmacological targeting of HDACs has high potential. However, most HDAC inhibitors used for clinical trials lack specificity which might contribute to side effects. Despite many years of intense research, surprisingly little is known about the substrate specificity of individual HDAC enzymes. To elucidate the consequences of more specific targeting of individual HDAC isoforms, detailed analysis of the catalytic function of each enzyme is required. Albeit HDACs being well studied in general, cross-comparison between the studies is difficult due to the use of different model systems and the frequent use of inhibitors, which lack specificity. Also, commonly used knockout or knockdown models abolish the structural function of the enzymes, potentially not accurately reflecting the situation of inhibitor treatment which only causes enzymatic inactivation. We suggest that the use of catalytic inactive mutants instead of knockouts might be another step towards mimicking isoform specific enzyme inhibition and provide a detailed comparative, side-by-side analysis of class I HDACs.

## Introduction

Histone deacetylases (HDACs) are important cellular regulators of chromatin structure and gene expression. HDACs can function as enzymes that remove acetyl groups from acetyl-lysines of histones. Through this activity, HDACs can either repress or activate gene expression [1–4]. The deacetylation of non-histone targets [5] can impact protein stability, interactions, localization or molecular functions, adding another level of regulatory complexity [6]. Mammalian lysine deacetylases are grouped into 4 classes. The class I HDACs comprise homologues of the yeast Rpd3 deacetylase and include the Zn^2+^-dependent enzymes HDAC1, HDAC2, HDAC3 and HDAC8 [7–9]. The HDAC1 and HDAC2 loci arose from a gene duplication [10] and show the closest homology among class I HDACs [8,9,11]. HDAC1 and HDAC2 are nuclear enzymes and represent the catalytic core of the same multiprotein complexes, including the SIN3, NURD and COREST complexes [11–14]. HDAC3 and HDAC8 are found both in the nucleus and the cytoplasm [15,16]. HDAC3 is the catalytic subunit of NCOR/SMRT co-repressor complexes [17] but also interacts with class IIa HDACs to regulate gene expression [18]. HDAC8 forms homodimers [19] and seems to exert its function without being part of a multiprotein complex [16].

Full body class I HDAC deletions are lethal in mice indicating that these enzymes are essential players in development and cell survival [20–26]. In contrast, HDAC1 and HDAC2 are partially redundant and able to compensate for each other in cell type-specific deletion analysis. Other HDACs are less likely to compensate for loss of the HDAC3 or HDAC8 enzyme (reviewed in [8]).

Class I HDACs are overexpressed in a variety of human cancers, including prostate cancer [27], bladder cancer [28], gastric and ovarian cancers [29,30], breast cancer [31] and neuroblastoma [32]. Ablation of class I HDACs results in reduced proliferation of tumor cells [19,33,34]. Aberrant histone modification patterns can lead to increased expression of oncogenes or downregulation of tumor suppressors [35]. Moreover, the function of many non-histone proteins is associated with cancer development and cell proliferation. Indeed, the acetylation status of the tumor suppressor p53, the oncogene c-MYC and the acetyltransferase p300 impacts their functional state [36].

Previous studies have contributed enormously to characterizing individual class I HDACs, but cross-comparison between studies is difficult due to the use of different model systems, different conditions and possible redundancy between HDACs [20,26,34,37–41]. In addition, it is unclear whether knockout models reflect the effects of HDAC inhibitors (HDACis) since genetic deletion of enzymes may impact non-catalytic functions [42–45]. A recent study has demonstrated a dominant negative (DN) effect of inactive HDAC2 on mouse development and corepressor complex activity, effects not seen upon HDAC2 deletion [46]. In short, side-by-side analysis of individual HDAC inactivation would provide much-needed insight into the catalytic functions of individual enzymes.

Small molecule inhibitors of HDACs induce a range of antitumor responses including cell death, cell cycle arrest, cellular senescence, terminal differentiation, autophagy or enhanced immunogenicity in tumor cells [47]. Five HDACis are FDA-approved for anticancer therapy and additional drugs are being tested in clinical trials [48]. While HDACis have limited effects when administered alone, combinatorial administration with tyrosine kinase inhibitors, proteasome inhibitors, immunomodulators or radiotherapy shows promising results [49]. Most currently used HDACis are pan-HDACis that act on all HDAC classes (not including sirtuins) [50] and cause a variety of side effects. More specific HDACis may decrease the risk of side effects and increase treatment benefits ([13,51,52].

To systematically compare class I HDACs in terms of histone deacetylase activity, cellular functions, and regulation of the acetylome and transcriptome, we used a toolbox of genetically modified HAP1 cells as a model system. This cell line was originally derived from the human chronic myelogenous leukemia cell line KBM7 [53] and genetic targeting is simplified as HAP1 cells are haploid [54]. We compared cell lines which express individual catalytically inactive, but structurally intact, HDAC isoforms in the presence or absence of the respective endogenous enzyme. We hypothesized that these cell lines mimic the partial or full pharmacological inhibition of individual class I HDACs. Therefore, we included class I HDACis in our experiments to test whether their effects correlate with the effects of the catalytically inactive mutants of specific class I HDACs.

Inactivation of HDAC1, HDAC2 and HDAC3, the three class I members with significant histone deacetylase activity, led to specific changes in the acetylome. Cells expressing dominant negative HDAC1 or HDAC2 showed overlapping increases of acetylation patterns of histones and multiple chromatin-associated factors. Differently, HDAC3 deacetylation targets were associated with a broad range of cellular functions, also apart from chromatin-related processes. Similarly, analysis of the transcriptome revealed that different gene sets are derepressed following HDAC1/2 inactivation and HDAC3 inactivation. In particular HDAC1/2 inactivation resulted in considerable overlaps to MS-275 induced effects. Finally, we propose that the specific, catalytic inactivation of individual class I HDACs might be sufficient to sensitize cells to anticancer drugs. The use of inactive HDACs might be advantageous for the identification of relevant target HDACs in future clinically relevant screens and in mouse disease models.

## Materials and methods

### Cloning of constructs for targeting the *AAVS1* locus

The pVJV vector was created as an adapted version of the pUC57_EF1A_AAVS1 shuttle vector (Haplogen/Horizon Discovery). Via oligoadapters (SalSrfXbaEco_F: TCG ACG CCC GGG CAT TGT ATT CGT CTA GAG, SalSrfXbaEco_R: AAT TCT CTA GAC GAA TAC AAT GCC CGG GCG), we inserted SrfI/XbaI cutting sites into the multiple cloning sites. ORFs of genes of interest (GOI) *Hdac1 wt/ Hdac1 H141A* (mouse), *Hdac2 wt/ Hdac2 H142A* (mouse), *Hdac3 wt/ Hdac3 H135A* (human) were amplified with primers containing the SrfI/XbaI cutting sites (AAC CCT CAC TAA AGG GAA CAA AAG CTG GAG and CCA TCT AGA TTG GGT ACA CTT ACC TGG TAC). Using XbaI and SrfI restriction enzymes (NEB) and the Quick Ligation Kit (NEB), the GOI with a C-terminal FLAG-tag was inserted downstream of the EF1a promoter and upstream of a bGH-polyA signal. The pVJV vector contains two homology arms up- and downstream of the *AAVS1* integration region (5´AAVS1-LHA and 3´AAVS1-RHA).

### Cell culture

HAP1 cells were grown in IMDM+++ medium (IMDM (12440053, Thermo Scientific), supplemented with Penicillin-Streptomycin (P4333, Sigma-Aldrich, 1% final concentration) and fetal bovine serum (F7524, Sigma-Aldrich, 10% final concentration)) at 37°C, 5% CO_2._ In case of inhibitor treatments, JQ12 [55,56] and MS-275 (S1053, Selleck Chemicals) were used at indicated concentration and period of time.

### Generation of class I HDAC knockout cell lines

HAP1 cells were transiently transfected with expression plasmids for *Streptococcus pyogenes* pCas9 (#771) and suitable gRNAs using TurboFectin (OriGene) according to manufacturer’s instructions. A plasmid encoding a blasticidin-resistance gene was co-transfected and cells were selected transiently with 20 μg/ml blasticidin. Transfected cells were subjected to limiting dilution to obtain clonal HAP1 lines. The indel introduced by CRISPR/Cas9 was verified by PCR and Sanger sequencing.

gRNAs:

HDAC1: TGA GTC ATG CGG ATT CGG TG

HDAC2: TGG GTC ATG CGG ATT CTA TG

HDAC3: TCT TAT AGA GAC CGT AAT GC

HDAC8: AAT CAA AGA ATG CAC CAT AC

### Targeting of the *AAVS1* locus

Cells were transfected, selected and subjected to limiting dilution similar as described above. Transfection was performed with a gRNA expression plasmid (sequence of gRNA: GTC ACC AAT CCT GTC CCT AG) targeting the *AAVS1* locus, and the AAVS1 donor vector (pVJV) harboring the GOI.

### Viability assay

Wildtype and mutant HAP1 cell lines were seeded into triplicate wells of solid white 96-well plates (2000 cells per well) and allowed to attach overnight. Cell viability was measured using CellTiter-Glo Luminescent Cell Viability Assay and Glomax Discover plate reader (Promega). In case of growth curves, the measurement was performed every 24 hours, starting from the day after seeding, for 4 time points (-24, 0, 24, 48 hours). JQ12 or MS-275 treated cells (0.5, 1.5 or 3 μM final concentration) were analyzed 72 hours after treatment start.

### Apoptosis analysis

5×10^6^ cells were collected 48 hours after seeding, fixed for 20 min in 2% paraformaldehyde (Merck Life Science) and for 30 min in 75% ethanol. Cells were permeabilized for 10 min with 0.1% TritonX100 (Merck Life Science), blocked for 30 min in 10% donkey serum (Merck Life Science) and then incubated for 1 hour with 1:50 diluted anti-cleaved caspase 3 antibody in phosphate-buffered saline (PBS) (Cell Signaling, #9661). Cells were then washed and incubated for 1 hour with 1:400 diluted Alexa 488 anti-rabbit secondary antibody (Jackson Immuno Research #751-545-152). Samples were acquired using FACSCelesta flow cytometer (BD Bioscience) and data were analyzed using FlowJo software 10.6.1 (BD Bioscience).

### DNA damage analysis

DNA damage was measured by immunofluorescence as described [39,57]. Briefly, cells were grown on coverslips, washed with PBS, fixed in 3.7% formalin for 10 min and then permeabilized with 0.5% Triton-X-100 in PBS for 3–5 min at room temperature (RT). Cells were blocked with 2.5 ml of 10% normal goat serum (Sigma) for 30 min and stained with mouse anti-γH2AX antibody (Millipore, 05–636) for 1 hour at RT. Cells were then incubated with a 1:600 dilution of the secondary goat anti-mouse Alexa 488 antibody (Thermo-Life Technologies, A11029) for 45 min at RT. Hoechst 33258 (Sigma, 1:1000) staining was performed to visualize the nuclei. Images were captured using a Zeiss Axioskop Mot plus microscope and analyzed using the AxioVision software. For quantitation, γ-H2AX foci in at least 100 cells were counted.

### Sensitization assay using HAP1 cells

Compounds dissolved in dimethyl sulfoxide (DMSO) at 1000x the final assay concentration were transferred to clear 384-well plates using acoustic compound transfer (Labcyte Echo 550). Each plate contained 320 compound wells, 32 negative control wells with equal concentration of DMSO and 32 positive control wells (bortezomib, 10 μM (primary screen) or 13.5 μM (secondary screen)). In an initial screen, 105 approved cancer drugs [58] were screened in triplicate using an 8-point dose response analysis against indicated cell lines and inhibitor-treated cells (HAP1 wildtype cells treated with 1 μM MS-275 or 1 μM JQ12). In case of HDAC inhibitor (HDACi) treatment, cells were pre-treated for 6 hours before incubation with the cancer drugs. Cells were seeded onto compound-containing wells and ATP levels were measured after 72 hours as surrogate of cell viability (Promega CellTiterGlo). Viability data were normalized to plate-internal mean negative control well signals (set to 100% viability) and plate-internal mean positive control well signals (set to 0% viability) after outlier removal. In a follow-up screen, hits from the initial screen were retested in 8-point dose response in duplicates. The starting concentrations of shown treatments were as following: 4SC-202: 27 μM, decitabine: 67.5 μM, alisertib: 13.5 μM, axitinib: 27 μM. Starting concentrations were titrated as 1:3 dilution series among 7 further wells and the second screen was performed similar as described for the first screen.

### Sensitization assay using squamous cell carcinoma cell lines

Half maximal inhibitory concentration (IC50) values for MS-275, alisertib, decitabine and sorafenib were defined by seeding head and neck squamous cell carcinoma (HNSCC) cell lines at the appropriate density (UM-SCC18 at 4000 cells/well; UM-SCC23, UM-SCC104 and UPCI-SCC152 at 5000 cells/well) in 96-well plates. Twenty-four hours later cell lines were treated with either vehicle or different concentrations of drugs (MS-275 starting from 15 μM for 5 serial 1:2.5 dilutions; decitabine starting from 250 μM for 5 serial 1:2 dilutions; sorafenib starting from 33.33 μM for 5 serial 1:1.5 dilutions; alisertib starting from 40 μM for 5 serial 1:1.5 dilutions) for 72 hours. IC50 values were evaluated also for drug combinations (MS-275 in combination with alisertib, decitabine or sorafenib) at the same concentrations described above. HNSCC cell lines were seeded in 96-well plates and treated 24 hours later with vehicle or different concentrations in all possible combinations of the two drugs for 72 hours. Cell proliferation was assayed with CellTiter-Glo Luminescent Cell Viability Assay, following the manufacturer’s instructions.

### RNA isolation

Total RNA was isolated using Trizol reagent (Thermo Fisher Scientific) as described in the manual. The isolated RNA, dissolved in nuclease-free ddH_2_O, was then subjected to another precipitation step by adding 1/10 vol. 3 M sodium-acetate (pH 5.2) and 2.5 vol. 96% ethanol. Following overnight-incubation, the samples were centrifuged (12000 rpm, 30 min, 4°C) and washed with 75% ethanol. Pellets were air-dried and dissolved in a suitable amount of nuclease-free ddH_2_O (shaking at 300 rpm, 10 min, 55°C). The RIN values of the RNAs used for RNA-seq were between 8.0 and 10.0.

### RNA-sequencing

#### NGS library preparation

Total RNA was quantified using the Qubit 2.0 Fluorometric Quantitation system (Thermo Fisher Scientific, Waltham, MA, USA) and the RNA integrity number (RIN) was determined using the Experion Automated Electrophoresis System (Bio-Rad, Hercules, CA, USA). RNA-seq libraries were prepared with the TruSeq Stranded mRNA LT sample preparation kit (Illumina, San Diego, CA, USA) using Sciclone and Zephyr liquid handling workstations (PerkinElmer, Waltham, MA, USA) for pre- and post-PCR steps, respectively. Library concentrations were quantified with the Qubit 2.0 Fluorometric Quantitation system (Life Technologies, Carlsbad, CA, USA) and the size distribution was assessed using the Experion Automated Electrophoresis System (Bio-Rad, Hercules, CA, USA). For sequencing, samples were diluted and pooled into next generation sequencing (NGS) libraries in equimolar amounts.

#### Next generation sequencing and raw data acquisition

Expression profiling libraries were sequenced on HiSeq 3000/4000 instruments (Illumina, San Diego, CA, USA) following a 50-base-pair, single-end recipe. Raw data acquisition (HiSeq Control Software, HCS, HD 3.4.0.38) and base calling (Real-Time Analysis Software, RTA, 2.7.7) was performed on-instrument, while subsequent raw data processing off the instruments involved two custom programs based on Picard tools (2.19.2; https://github.com/epigen/picard/, https://broadinstitute.github.io/picard/). In a first step, base calls were converted into lane-specific, multiplexed, unaligned BAM files suitable for long-term archival (IlluminaBasecallsToMultiplexSam, 2.19.2-CeMM). In a second step, archive BAM files were demultiplexed into sample-specific, unaligned BAM files (IlluminaSamDemux, 2.19.2-CeMM).

#### Transcriptome analysis

NGS reads were mapped to the Genome Reference Consortium GRCh38 assembly via “Spliced Transcripts Alignment to a Reference” (STAR) [59] utilizing the “basic” Ensembl transcript annotation from version e100 (April 2020) as reference transcriptome. The hg38 assembly flavor of the UCSC Genome Browser was preferred for downstream data processing with Bioconductor packages. Therefore, for entirely technical reasons, Ensembl transcript annotations had to be adjusted to UCSC Genome Browser sequence region names. STAR was run with options recommended by the ENCODE project. Aligned NGS reads overlapping Ensembl transcript features were counted with the Bioconductor (3.14) GenomicAlignments (1.30.0) package via the summarizeOverlaps function in Union mode. Given that the Illumina TruSeq stranded mRNA protocol leads to sequencing of the first strand, all reads needed inverting before counting. Transcript-level counts were aggregated to gene-level counts and the Bioconductor DESeq2 (1.34.0) package [60] was used to test for differential expression based on a model using the negative binomial distribution.

An initial exploratory analysis stage was based on a matrix of read counts after DESeq2 variance stabilizing transformation. A principal component analysis (PCA, R 4.1.3 stats prcomp) was based on the transformed count of the top 500 most variable genes (Bioconductor genefilter, 1.76.0), only. The color scale of a sample distance heatmap encodes the Euclidean distance of transformed counts (R 4.1.3 stats dist), while the dendrogram represents a hierarchical cluster analysis (R 4.1.3 stats hclust) via the complete linkage method.

Biologically meaningful results were extracted from the model, log2-fold values where shrunk with the CRAN ashr (2.2.-54) package, while two-tailed p-values obtained from Wald testing were adjusted with the Bioconductor Independent Hypothesis Weighting (IHW, 1.22.0) package [61,62]. The resulting gene lists were annotated, filtered for significantly differentially up- and downregulated genes and independently subjected to gene ontology analysis of biological processes (Enrichr: https://amp.pharm.mssm.edu/Enrichr/). Gene set enrichment analysis (GSEA, 4.2.3) [63,64] was performed with the expression data set based on DESeq2-normalized counts and the gene set permutation option. In a targeted GSEA, the publicly available gene sets “NRSF_01” (https://www.gsea-msigdb.org/gsea/msigdb/cards/NRSF_01) and “WP_NUCLEAR_RECEPTORS_METAPATHWAY” (https://www.gseamsigdb.org/gsea/msigb/cards/WP_NUCLEAR_RECEPTORS_METAPATHWAY.html) were used.

The RNA-seq datasets have been deposited to the NCBI Gene Expression Omnibus (GEO) under GSE193885 (https://www.ncbi.nlm.nih.gov/geo/query/acc.cgi?acc=GSE193885).

#### Protein isolation

Cell pellets were resuspended in Hunt protein extraction buffer (20 mM Tris-HCl pH 8.0, 100 mM sodium chloride, 1 mM ethylenediamine tetraacetic acid (EDTA), 0.5% NP-40), supplemented with cOmplete protease inhibitor cocktail (Roche), 100 μM phenylmethylsulfonyl fluoride (PMSF), 10 mM sodium fluoride, 10 mM β-glycerophosphate, 10 μM sodium molybdate and 100 μM orthovanadate. Following two freeze-and-thaw cycles and full speed centrifugation, the supernatant containing extracted soluble protein was further used. Protein concentration was determined using Bio-Rad Protein Assay Dye Reagent Concentrate (5000006, Bio-Rad).

#### Immunoprecipitation

600 μg—1 mg of protein extract was incubated with the appropriate antibody for 1 hour. 4 μg of following polyclonal antibodies were used: COREST (07–455, Millipore), HDAC8 (ab187139, Abcam). In case of HDAC1/2/3 immunoprecipitations (IPs), 30 μl of monoclonal antibodies were used: HDAC1 (clone 10E2), HDAC2 (clone 3F3), HDAC3 (clone 3G6) (all generated in collaboration with Egon Ogris, Medical University of Vienna).

For IP samples, 30 μl of protein A- or protein G-beads (10002D or 10004D Dynabeads, Thermo Fisher Scientific) were prepared, washed three times in Hunt buffer, blocked in 1 mg/ml bovine serum albumin (BSA) for 1 hour at 4°C and incubated with the protein extract overnight. The next day, the beads were washed 3x with Hunt buffer.

For FLAG immunoprecipitation, 30 μl of anti-FLAG M2 Magnetic Beads (M8823, Sigma-Aldrich) were used and after 3 washes in 1x Tris-buffered saline (TBS), the beads were blocked for 30 min in 1 mg/ml BSA. The beads were incubated with 80 μg—1 mg of protein overnight at 4°C. The next day, the beads were washed 3x with 1x TBS.

After the last wash, 2/3 of the IP sample were used for Western blotting and 1/3 of the IP sample was used for the histone deacetylase assay. For all immunoprecipitations, controls to check background with the corresponding amounts of extract were included.

#### Acetyl(K) immunoprecipitation

Per sample, 27 μl of anti-acetyl lysine antibody, immobilized to agarose beads (ICP0388, Immunechem) were washed with PBST (PBS containing 0.1% Tween-20) three times (1000 rpm, 3 min, 4°C), followed by two washes with 0.1 M NaH_2_PO_4_, 1 M NaCl and one wash with PBST. 1 mg of protein extract of untreated or 24 hour MS-275 treated wildtype cells (3 μM) was added and incubated for 2 hours, rotating overnight at 4°C. The beads were washed with PBST for four times and bound proteins were eluted for immunoblot analysis, as described below.

#### Immunoblot analysis

20 μg of whole cellular protein were denatured in sodium dodecyl sulfate (SDS) loading dye as input. For IP samples, the washing buffer was completely removed and proteins were eluted from the beads with SDS loading dye (without dithiothreitol (DTT)) for 5 min at 55°C. The eluate was mixed with SDS loading dye (with DTT) and denatured. Proteins were separated by SDS-polyacrylamide gel electrophoresis and transferred onto a nitrocellulose membrane (Amersham Protran, GE10600001, Sigma-Aldrich) by the wet transfer method. The membrane was blocked in blocking solution (1x PBS, 1% polyvinylpyrrolidone, 1% non-fat dried milk, 0.1% Tween-20, 0.01% sodium azide, pH 7.4) and incubated with following primary antibodies: FLAG M2 (F1804, Sigma), HDAC1 (polyclonal rabbit SAT208, Seiser lab), HDAC2 (ab7029, Abcam), HDAC3 (ab7030, Abcam), HDAC8 (sc11405, Santa Cruz), COREST (RCOR1, 07–455, Millipore), β-actin (ab8226, Abcam or ab8227, Abcam), vinculin (13901S, Cell signaling). ECL Western blotting detection reagents (RPN2106, GE Healthcare) were used for detection together with a FUSION FX chemiluminescence imaging system.

#### Deacetylase activity assay using histones

The deacetylase assay was conducted on 20 μg of total cellular extract or on beads carrying immunoprecipitated proteins in 20 μl total volume as described previously [65,66]. 4 μl of [^3^H]-acetate-labeled chicken reticulocyte core histones (1.5–2 mg/ml) were added. Following incubation for 1 hour (total cellular extract samples) or 5 hours (immunoprecipitation samples) on a thermomixer (300 rpm, 30°C), 35 μl of Histone Stop Solution (1 M HCl, 0.4 M sodium acetate) and 800 μl of ethyl acetate were added. After vortexing for 15 sec, the samples were centrifuged with a swing out bucket centrifuge (10000 rpm, 4 min, RT). 600 μl of the organic phase were mixed with 3 ml of Scintillation Solution (5 g/l PPO, 0.5 g/l POPOP, in toluene). Counts per minute (CPM) were determined with a Liquid Scintillation Analyzer (Packard).

### Mass spectrometry

#### Preparation of cells

Three biological replicates of each HAP1 cell line and wildtype cells treated with MS-275 (3 μM for 6 or 24 hours) were prepared. Cells were washed twice in 1x PBS, supplemented with 10 mM sodium butyrate (303410, Sigma-Aldrich) and 3 mM nicotinamide (NAM) (72340 Sigma-Aldrich), and pelleted prior to lysis.

#### Cell extract and mass spectrometry sample preparation

2x10^7^ cells per replicate were prepared in lysis buffer containing 8 M urea, 50 mM Tris-HCl pH 8.0, 150 mM NaCl, 1 mM PMSF, 5 mM sodium butyrate, 10 ng/ml NAM, 10 ng/ml trichostatin A (TSA) and 1x cOmplete protease inhibitor cocktail (+EDTA). 250 U of benzonase (Merck) were added to each sample. Lysis was achieved using a Bioruptor sonication device (Diagenode) applying 5 alternating cycles of 30 sec sonication and 30 sec cooling. Power level was set to H. Following lysis, cells were centrifuged at 15,000 rcf for 10 min at 4°C to remove insoluble material. The protein fraction was precipitated by addition of 4x volumes of cold (-20°C) 100% acetone and 4°C incubation overnight. The protein pellet was washed with cold (- 20°C) 80% acetone, air dried for 5 min, and resolved in 8 M urea, 50 mM ammonium bicarbonate buffer (ABC). Protein concentration was determined using the Pierce 660 nm Protein Assay (Thermo Scientific). Proteins were reduced with 10 mM DTT for 45 min at RT, and subsequently carbamidomethylated with 20 mM iodoacetamide for 30 min at RT in the dark. The alkylation reaction was quenched by adding 5 mM DTT for 10 min. Prior to digestion, the urea concentration was reduced to 4 M with 50 mM ABC. Samples were predigested for 90 min at 37°C with Lys-C (Wako Laboratories) at an enzyme-to-substrate ratio of 1:100. Samples were digested overnight at 37°C with sequencing grade trypsin (Promega) at an enzyme-to-substrate ratio of 1:50. Following digestion, samples were acidified using 10% TFA (trifluoroacetic acid) (Sigma-Aldrich) to a final concentration of 0.5%, and desalted on a 50 mg tC18 Sep-Pak cartridge (Waters). Peptide concentrations were determined and adjusted according to UV chromatogram peaks obtained with an UltiMate 3000 Dual LC nano-HPLC System (Dionex, Thermo Fisher Scientific), equipped with a monolithic C18 column (Phenomenex). Desalted samples were dried for 30 min in a SpeedVac concentrator and subsequently lyophilized overnight.

#### Isobaric labeling using TMTpro 16plex

300 μg of trypsin-digested and desalted peptides were used for each sample for isobaric labeling. Lyophilized peptides were dissolved in 20 μl 100 mM triethylammonium bicarbonate (TEAB) (Sigma). 20 μg of peptides in TEAB from each sample and replicate were additionally mixed (final volume 60 μl) as reference sample (REF). 500 μg of each TMTpro16plex reagent (Thermo Fisher Scientific) were dissolved in 30 μl of 100% anhydrous acetonitrile and added to the peptide/TEAB mixes. The REF sample was mixed with 1.5 mg (90 μl) of TMTpro16plex reagent 134N and subsequently split in 3 aliquots. Labels used: 126C: wildtype; 127C: MS-275 6 hour; 128C: MS-275 24 hour; 129C: WT HDAC1 CI; 130C: WT HDAC2 CI; 131N: HDAC1 KO; 131C: WT HDAC3 CI; 132N: HDAC2 KO; 132C: WT HDAC8 CI; 133N: HDAC3 KO; 133C: REF; 134N: HDAC8 KO. Samples were labeled for 60 min at RT. 0.1 μl of each sample were pooled, mixed with 10 μl 0.1% TFA and analyzed by mass spectrometry (MS) to check labeling efficiency. For quenching, 5 μl of 5% hydroxylamine (0.4% final concentration) were added and the reaction was incubated for 25 min at RT. Samples were pooled and subsequently desalted with Sep-Pak tC-18 (200 mg) cartridges (Waters). Desalted samples were dried for 30 min in a SpeedVac vacuum centrifuge and subsequently lyophilized overnight.

#### Neutral pH fractionation

Peptides were dissolved in 80 μl 10 mM ammonium formate, pH 6.8 and separated on a UltiMate 3000 Dual LC nano-HPLC System (Dionex, Thermo Fisher Scientific) equipped with a XBridge Peptide BEH preparative C18 (130 Å, 3.5 μm, 4.6 mm x 250 mm) column (Waters). A maximum of 5 mg was loaded. Peptides were separated using a 60 min gradient of 5 to 50% acetonitrile in 10 mM ammonium formate with a flow rate of 1.0 ml min^−1^. 45 fractions were collected and subsequently pooled in a noncontiguous manner into 8 pools. For example, to generate the first pool, original fractions 1, 9, 17, 25, 33 and 41 were combined. Pools were dried for 30 min using a SpeedVac concentrator and subsequently lyophilized overnight.

#### Acetylated peptide enrichment

Acetylated peptides were enriched using the PTMScan Acetyl-Lysine Motif Kit (Cell Signaling). Each lyophilized peptide pool was dissolved in 175 μl of 1x PTMScan IAP buffer. Aliquots of roughly 10 μg of peptide/pool were removed for proteome analysis. Antibody beads (one vial of antibody-bead slurry per tandem mass tag (TMT) multiplex setup (~4.8 mg of peptide)) were washed four times with 1x PBS and split into eight aliquots. Peptides resuspended in 1x IAP were added and the mix was incubated for 2 hours at 4°C with end-over-end rotation. Following enrichment, PTMScan antibody beads were washed twice in 1x IAP and subsequently three times with high pressure liquid chromatography (HPLC)-grade water (Fisher Chemical). Peptides were eluted twice from antibody beads with 20 μl of 0.15% TFA, respectively. Additional 5 μl of 0.15% TFA were used to wash out residual peptides. Eluates were united and desalted using two C18 StageTips per pool as described in [67]. Enriched peptides were eluted twice with 25 μl of 40% acetonitrile, 0.1% TFA. Eluted peptides were dried to completeness in a SpeedVac concentrator and subsequently resuspended in 0.1% TFA.

#### Mass spectrometry measurements

LC-MS/MS analysis was performed on an UltiMate 3000 Dual LC nano-HPLC System (Dionex, Thermo Fisher Scientific), containing both a trapping column for peptide concentration (PepMap C18, 5x 0.3 mm, 5 μm particle size) and an analytical column (PepMap C18, 500x 0.075 μm, 2 μm particle size, Thermo Fisher Scientific), coupled to a Q Exactive HF Orbitrap (with HCD, higher-energy collisional dissociation mode) mass spectrometer (Thermo Fisher) via a Proxeon nanospray flex ion source (Thermo Fisher). For peptide separation on the HPLC, the concentration of organic solvent (acetonitrile) was increased from 1.6% to 32% in 0.1% formic acid at a flow rate of 230 nl/min, using 2 hours gradient time for acetylome analysis and 3 hours gradient time for proteome analysis. The instruments were operated in data-dependent acquisition (DDA) mode with dynamic exclusion enabled. MS1 survey scans were recorded with the following parameters: resolution 120,000, scan range 375–1,500 m/z, automatic gain control (AGC) target 3×10^6^, and maximum injection time (IT) 50 ms. The top 20 precursors were selected for MS2 analysis (HCD) with the following parameters: resolution 45,000, AGC 2×10^5^, maximum IT 96 ms (proteome) or 200 ms (acetylome), isolation window 0.7 m/z, and normalized collision energy (NCE) 35. The minimum AGC target was set at 8×10^3^ (acetylome) or 1×10^4^ (proteome), which corresponds to an intensity threshold of 4×10^4^ or 1×10^5^, respectively. In addition, unassigned, singly and >6+ charged species were excluded from MS2 analysis and dynamic exclusion was set to 20 or 30 sec, respectively.

#### Mass spectrometry data analysis with MaxQuant

Raw MS data were analyzed using MaxQuant [68] software version 1.6.17.0, using default parameters with the following modifications. MS2 spectra were searched against the Homo sapiens (human) Uniprot database (release 2020.01; with isoforms) and a database of common laboratory contaminants (provided with MaxQuant). Enzyme specificity was set to “Trypsin/P”, the minimal peptide length was set to 7 and the maximum number of missed cleavages was set to 4. Carbamidomethylation of cysteine was searched as a fixed modification. “Acetyl (Protein N-term)”, “Oxidation (M)” and “Acetyl (K)” were set as variable modifications. A maximum of 6 variable modifications per peptide was allowed. Proteome and acetylome raw data were included in the same search but in separate parameter groups. For proteome measurements “Acetyl (K)” was not used as a variable modification and the maximum number of missed cleavages was set to 2. The identification and quantification information of acetylated peptides, sites, and proteins was obtained from the MaxQuant “Acetyl (K) Sites”, “ModificationSpecificPeptides”, and “ProteinGroups” tables. The data have been deposited to the ProteomeXchange Consortium [69] via the PRIDE partner repository with the dataset identifier PXD030164 (https://www.ebi.ac.uk/pride/archive/projects/PXD030164).

Data were analyzed in R (4.1.0) using custom scripts (**S1 Material**). The analysis procedure covered: correction for isotopic impurities of labels, within-plex median normalization, between-plex IRS-normalization [70], and statistical between-group comparisons using LIMMA (3.50). To account for differences of protein abundance on lysine acetylation-site level, site intensities were normalized to protein intensities before group comparisons with LIMMA.

#### Targeted mass spectrometry

Based on the data from the isobaric labeling experiment, we selected 34 histone peptides which showed increased acetylation sites upon HDAC inactivation to validate these using label-free targeted MS. For normalization to total protein level, 18 peptides that were not found to be modified (experimentally or according to UniProt) were additionally selected. Peptide selection was performed using Skyline [71]. Samples for the validation experiment included untreated wildtype cells and cells treated for 2, 6 or 24 hours with 3 μM MS-275. Following sample preparation, peptides were separated using a 60 min gradient (HPLC setup as described above). Parallel-reaction monitoring (PRM) data acquisition was performed using a scheduled method with 2–5 min windows for each target based on the retention time determined from a DDA LC-MS/MS run (analyzed using MaxQuant as described above) of a test sample (conditions: wildtype HAP1, 3 μM MS-275, 24 hours). Raw data were obtained using an Orbitrap Q-Exactive HF-X (Thermo Fisher Scientific) mass spectrometer applying the following settings: survey scan with MS1 45 k resolution, AGC 3E6, 50 ms IT, over a range of 340 to 1000 m/z, PRM scan with 60 k resolution, AGC 2E5, 400 ms IT, isolation window of 0.7 m/z, and NCE of 28%. MS1 signals of indexed Retention Time (iRT) standards (Biognosys) were used as quality control for monitoring instrument performance in Skyline. Data analysis, manual validation of all transitions (based on retention time, relative ion intensities, and mass accuracy), and relative quantification was performed in Skyline. Three specific transitions were selected for each peptide and their peak areas were summed up for peptide quantification across charge states (total peak area). Unmodified peptides were used for calculating a normalization factor for each sample, which was finally used to normalize the intensities of modified peptides. We applied one-way ANOVA analysis to compare intensities from the mock samples to all inhibitor-treated samples.

Data of the targeted approach have been deposited to the PanoramaWeb [72] (https://panoramaweb.org/edvjs1.url, PXD030272).

#### Statistical analysis and data visualization

Heatmaps were created using python packages pyplot (Matplotlib), pandas, numPy and seaborn. GraphPad Prism version 5.00 for Windows (GraphPad Software, San Diego, California, USA) was used to generate bar graphs and growth curves and to determine IC50 values of drugs or drug combinations for SCC cell lines as described [73]. In case of HDAC assays, viability and DNA damage analysis, the analysis of two groups was performed using Welch‘s t-test, in case of three or more groups, One- or Two-way ANOVA analysis with Bonferroni multiple comparison testing was chosen. For OMICS data, the individual statistical analysis is indicated in corresponding method parts. Bands of Western blot images were cut out using Adobe Photoshop version 10.0 and figures were prepared using Adobe Illustrator version 16.0.0.

## Results

### Generation of a toolbox for comparative analysis of class I HDACs

**Fig 1:**
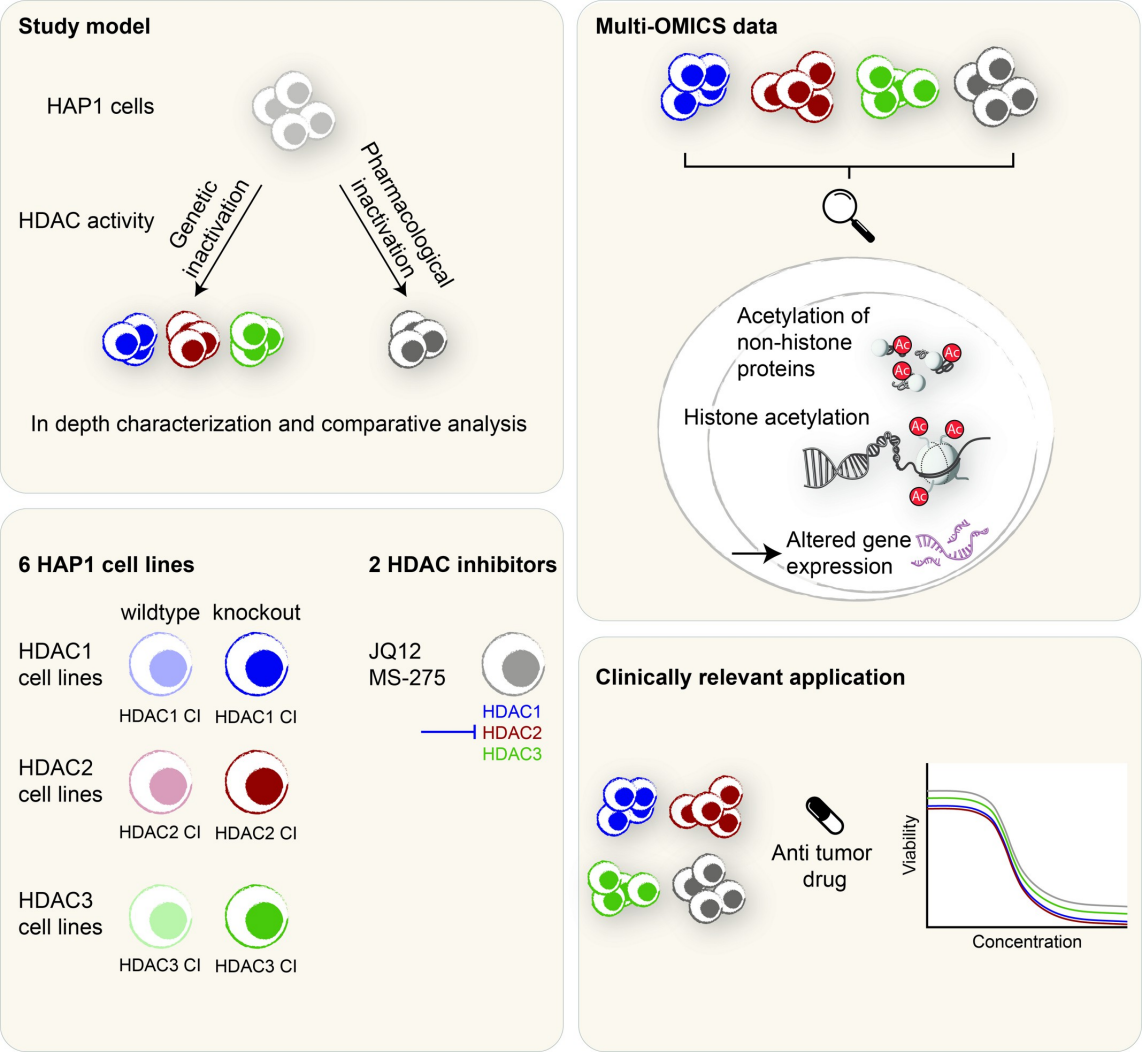
Study design. This study compared effects of genetic and pharmacological inactivation of HDAC1/2/3. Genetically modified cell lines expressing catalytically inactivate (CI) transgenic versions of HDAC1/2/3, in addition to cells treated with HDAC inhibitors, were used. The HDAC CI cell lines were investigated as potential novel tool to mimic partial or full HDAC inactivation. This was achieved by expressing transgenic HDAC1, HDAC2 or HDAC3 in the presence (wildtype background) or absence (knockout background) of the respective endogenous HDAC enzyme. We performed a comprehensive comparative approach using the HDAC1/2/3 CI cell lines and cells treated with the HDAC inhibitors JQ12 and MS-275. Based on global acetylome profiling and gene expression analysis, isoform-specific functions of HDAC1/2 and HDAC3 and similarities between genetic and pharmacological inactivation were investigated. A screening approach with anti-cancer drugs further addressed the question, if the HDAC1/2/3 CI model is suitable to study synergistic effects of isoform-specific HDAC inactivation.

To systematically address the catalytic functions of class I HDACs, we employed the nearly haploid human tumor cell line HAP1 [53,74] as model system. As starting point of our study, we determined the individual deacetylase activities of each of the class I HDAC enzymes. We immunoprecipitated HDAC1, HDAC2, HDAC3 and HDAC8 from wildtype (WT) HAP1 cells and used corresponding knockout (KO) cell lines as negative controls and measured the associated deacetylase activities with acetylated histones as substrate [65,66] (**S1A Fig**). Immunoprecipitated HDAC1, HDAC2 and HDAC3 displayed robust deacetylase activity, while HDAC8 was not active in this assay. The latter result is consistent with a previous report showing that HDAC8 prefers long chain fatty acyl lysines over acetylated lysine as substrate [75]. Based on this, we decided to further examine the effects of inactivating HDAC1, HDAC2 and HDAC3. Using pharmacological inhibitors and genetically manipulated cellular models (**Fig 1**), we wanted to determine if the specific catalytic inactivation of HDAC1, HDAC2 and HDAC3 enzymes causes different effects on cellular functions and reflects the effects of HDACi treatment. In detail, we performed a full comprehensive approach, including global lysine acetylome profiling and gene expression analysis, to compare inactive mutant expressing cells and cells treated with the class I HDAC is MS-275 and JQ12. The class I HDACi entinostat (MS-275) is a synthetic benzamide derivative with preference for HDAC1 and HDAC3 enzymes undergoing clinical trials for several types of cancer [76–78]. JQ12 is a recently developed class I HDACi with preference for HDAC1 and HDAC2 [56,79].

**Fig 2:**
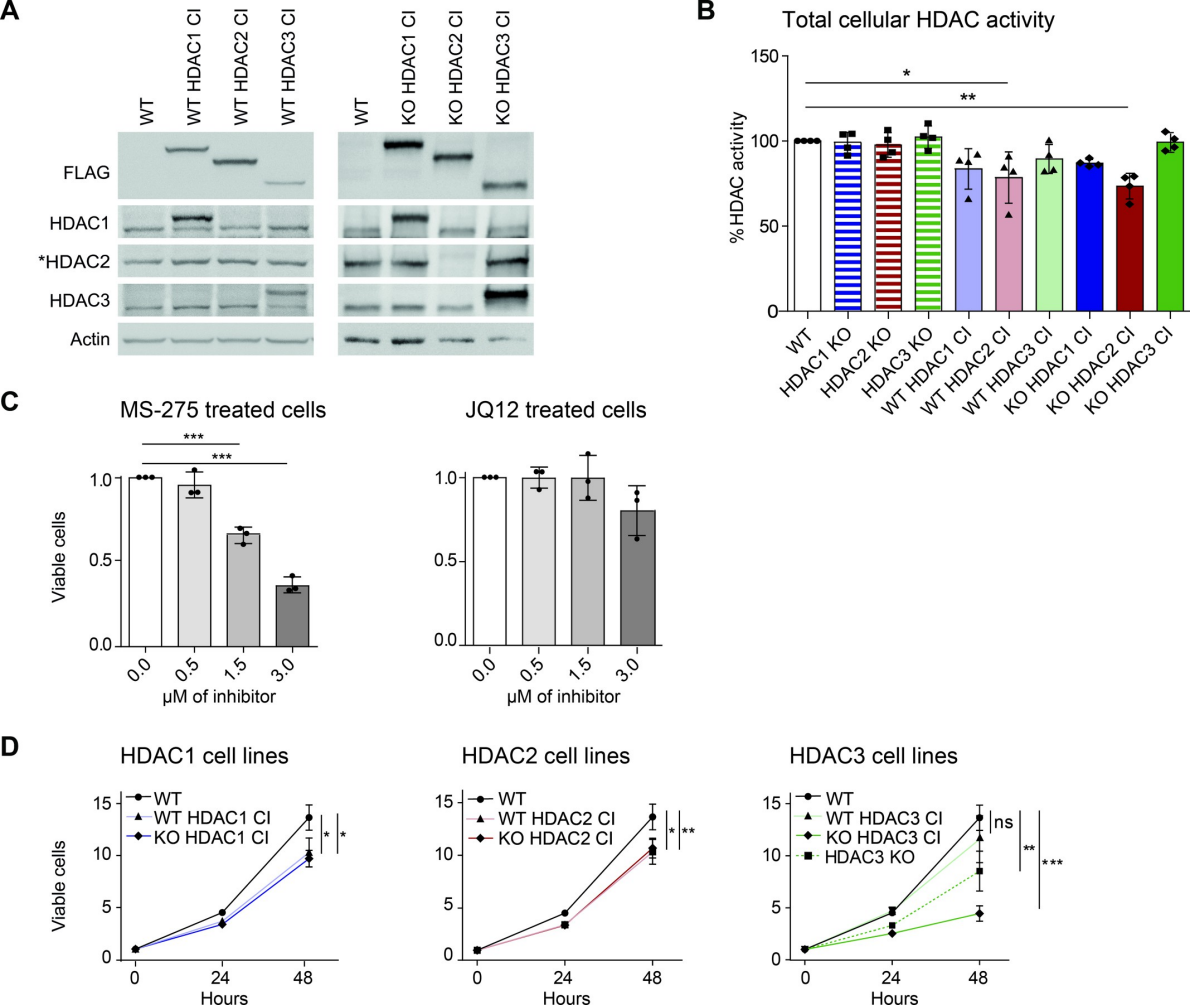
Cellular effects caused by HDAC1/2/3 inactivation. (A) Western blot analysis of HDAC1/2/3 CI transgene expression in the corresponding wildtype (WT) and knockout (KO) backgrounds. Antibodies specific for the FLAG epitope, HDAC1, HDAC2 and HDAC3 were used for detection. Beta-actin was used as loading control. *Note: FLAG-tagged HDAC2 is not recognized by the HDAC2 antibody. (B) Impact of HDAC deletion and inactivation on total cellular deacetylase activity towards histones. The data represent the mean values ± standard deviation (SD) of 4 biological replicates and significance was determined by one-way ANOVA. *p < 0.05, **p < 0.01. (C) Viability analysis of cells treated with increasing concentrations of MS-275 (left) and JQ12 (right) for 72 hours. (D) Proliferation of HDAC1/2/3 CI cells and HDAC3 KO cells measured over 48 hours. (C-D) Mean values ±SD of 3 biological replicates are shown. One-way ANOVA (C) or two-way ANOVA (D) was used to analyze significance. *p < 0.05, **p < 0.01, ***p < 0.001.

The inactivation of individual HDAC enzymes based on the mutation of a single conserved histidine residue in the catalytic center to alanine (HDAC1 H141A, HDAC2 H142A, HDAC3 H135A) [46,80,81]. We integrated the corresponding cDNAs, encoding C-terminally FLAG-tagged inactive HDAC enzymes, into the *AAVS1* safe harbor locus **(S1B Fig**). In our established cell lines, the CI mutants were either expressed in the presence or absence of endogenous wildtype enzyme (“WT HDAC CI” and “KO HDAC CI”, respectively) (**Figs 1 and 2A**). We hypothesized that these two scenarios would mimic partial or full inhibition of the respective HDAC.

Using cells expressing the transgenes in the respective KO background, we confirmed that the deacetylase activity was highly reduced in the HDAC CI mutants over HDAC WT transgenic control enzymes (**S1C Fig**). In addition, we compared the total cellular histone deacetylase activities of cells expressing the HDAC1/2/3 CI mutants to wildtype control cells and HDAC1/2/3 KO cells. Cells expressing HDAC1 CI in both wildtype or KO background showed a trend towards lower cellular activity, while expression of HDAC2 CI led to a significant reduction of total cellular histone deacetylase activity (**Fig 2B**). In contrast, HDAC3 inactivation or knockout of HDAC1, HDAC2 or HDAC3 had no effect. It was shown previously that expression of HDAC1 CI or HDAC2 CI causes dominant negative effects, potentially due to their incorporation into HDAC complexes [46].

### HDAC3 is crucial for cell viability

Class I HDACs have an important role in the control of cell growth and proliferation [8,9]. To determine the consequences of class I HDACis on cell viability, we treated wildtype cells with MS-275 or JQ12 for 72 hours (**Fig 2C**). Cell viability was significantly reduced upon MS-275 treatment at 1.5 μM or higher concentration, but not upon JQ12 treatment. Next, we analyzed how cell viability is affected upon expression of HDAC1/2/3 CI, either in the presence (WT) or absence (KO) of the respective endogenous wildtype enzyme (**Fig 2D**). The expression of HDAC1 CI and HDAC2 CI in both settings led to a slight but significant reduction. HDAC3 inactivation in the KO background strongly reduced cell viability, while the observed change for HDAC3 CI in WT cells was not significant. Therefore, we tested also the viability of HDAC3 KO cells, which was significantly reduced, confirming the crucial cellular function of HDAC3 (**Fig 2D**) [39,40,82]. Consistent with these results, inactivation of HDAC3 led to a significant increase in apoptosis (**S2A Fig**). Given the significant effects by additional HDAC3 ablation, we included HDAC3 KO cells in all further analyses. Since class I HDACs have a role in the maintenance of genome integrity [83,84], we additionally analyzed whether the inactivation of HDAC1/2/3 induces DNA damage. To this end, we quantified H2AX phosphorylation (γ-H2AX), a marker for DNA double strand breaks (**S2B and S2C Fig**). Inactivation or loss of HDAC3 led to a strong increase of γ-H2AX foci compared to wildtype cells. Taken together, catalytic inactivation of HDAC1 and HDAC2 causes a mild decrease in cell viability, whereas loss of HDAC3 and its activity leads to significant DNA damage, apoptosis and strongly reduced cell viability.

### HDAC1/2 or HDAC3 inactivation differently affects cellular lysine acetylation patterns

**Fig 3:**
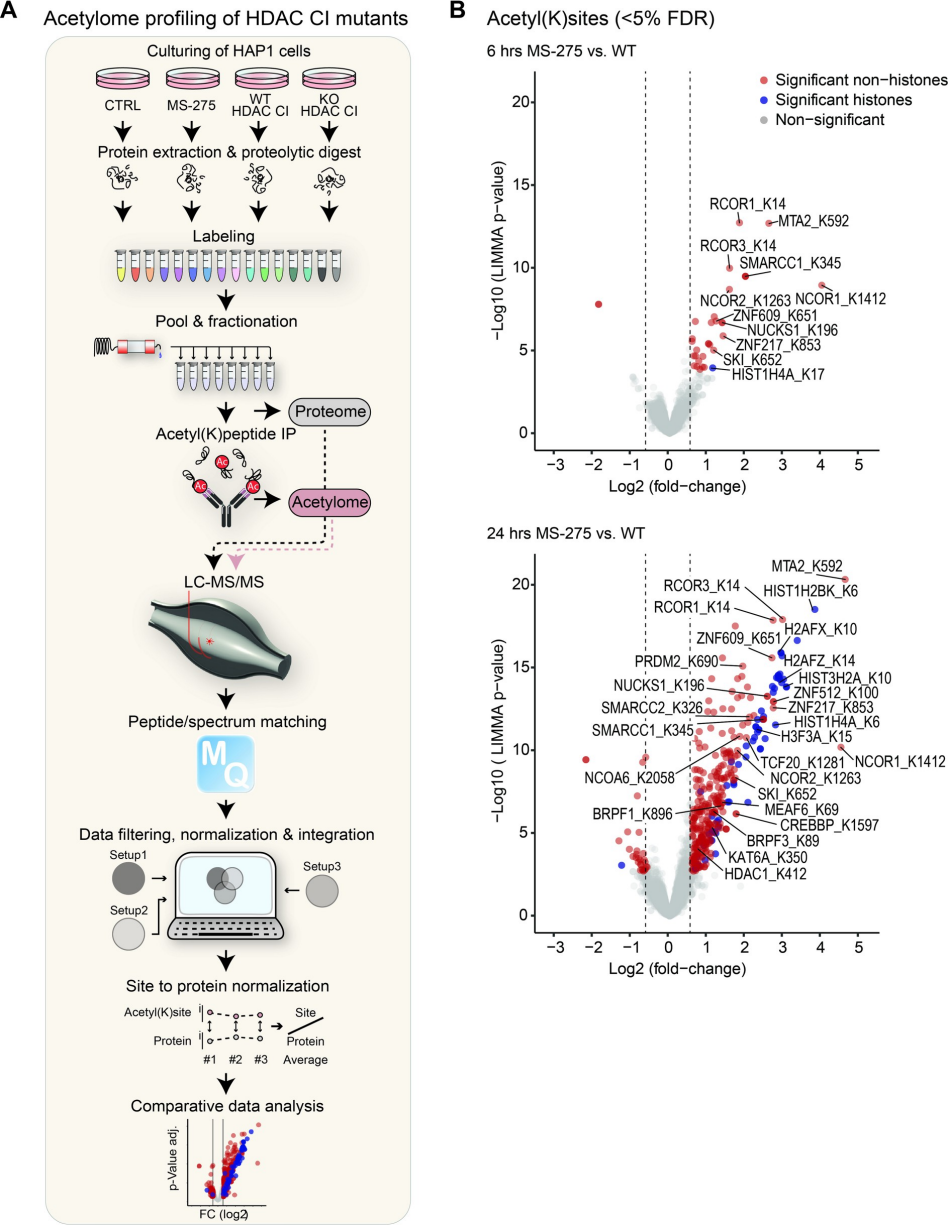
Acetylome analysis for cellular substrate identification of HDAC1/2/3. (A) Quantitative MS workflow. Cells were lysed and proteins were digested with trypsin. Peptides were labelled using TMTpro 16plex reagents, pooled, and fractionated using high pH HPLC. Aliquots for proteome measurements were removed. Acetylated peptides were purified by acetyl-lysine immunoprecipitation. Proteomes and acetylomes were analyzed by LC-MS/MS. Following data processing, acetylation sites were normalized to protein abundance and the effects of HDAC inactivation were assessed in a comparative analysis. (B) Volcano plots displaying global changes in lysine acetylation, induced by 6 hours (upper panel) and 24 hours (lower panel) of MS-275 treatment. Significantly regulated acetyl(K)sites (≥ 1.5-fold change over wildtype cells (dashed lines), padj-value ≤ 0.05) are highlighted in red (non-histone proteins) and blue (histones). Selected sites and gene names of corresponding proteins are annotated.

Previous studies have revealed changes in protein lysine acetylation upon pharmacological inhibition of HDACs [5,85,86]. However, the individual impact of HDAC1/2/3 catalytic function on the global acetylation pattern has, to our knowledge, not yet been systematically investigated in a single large-scale experimental (MS-shotgun) approach. Among class I HDACs, HDAC1/2/3 display a high catalytic activity and have an essential impact on total cellular HDAC activity (**S1A and S1C Fig** and **Fig 2B**) [20,44,87]. Therefore, we determined global lysine acetylome profiles upon HDAC1/2/3 inactivation in HAP1 cells and compared these patterns to those of HDACi-treated cells. In detail, we examined cells treated with MS-275 for 6 hours or 24 hours, and cells expressing catalytically inactive class I HDACs in the wildtype and KO background using quantitative mass spectrometry based on the recently developed TMTpro 16-plex isobaric labeling technology [88] (**Fig 3A** and **S1 Material**). In total, we identified 8,788 proteins and 33,968 acetylation sites, out of which 6,627 proteins and 13,187 acetylation sites could be reliably quantified in all three replicates. Changes in protein lysine acetylation sites were further normalized to the corresponding protein levels and are provided in **S1 Table**. Acetylation sites showing a fold change of ≥ 1.5 over wildtype cells and a padj-value of ≤ 0.05 were considered as significantly regulated.

Upon MS-275 treatment, the majority of proteins with affected sites (90%) showed an increase in acetylation (**Fig 3B**), which is the expected effect of deacetylase inactivation. Few non-histone targets, such as co-repressor proteins or other chromatin-associated factors, displayed elevated acetylation sites already after 6 hours of MS-275 treatment (22 hyperacetylated proteins) (**Fig 3B, upper panel**). However, 24 hours treatment evoked a further, large increase in acetylation on histones and non-histone targets (224 hyperacetylated proteins) (**Fig 3B, lower panel**). Proteins that were hyperacetylated upon MS-275 treatment show a large overlap with previously published HDAC inhibitor targets [5,85].

**Fig 4:**
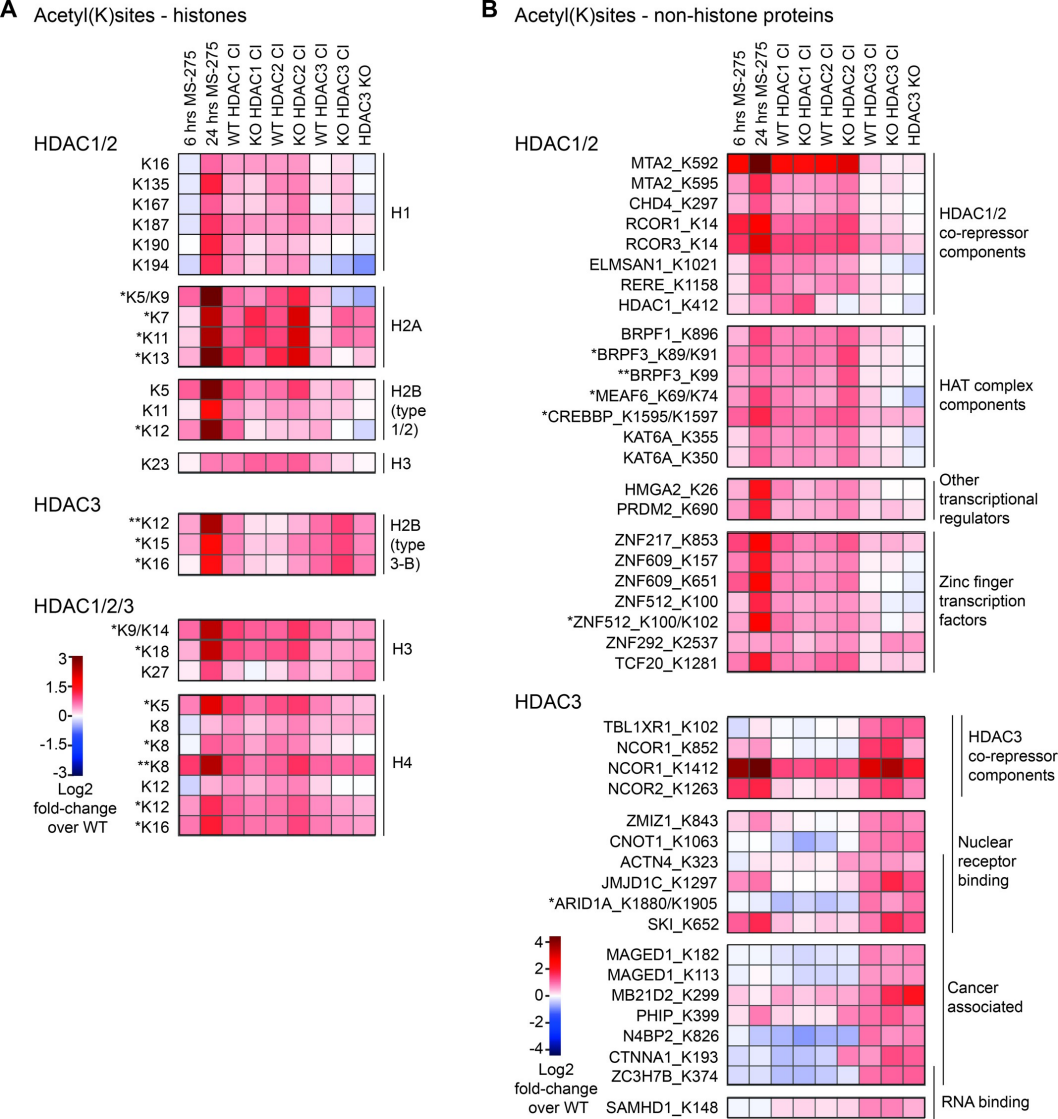
Histone and non-histone protein acetylation affected by HDAC1/2/3. (A-B) Heatmaps illustrating acetylation profiles (log2 fold change over wildtype) of a selected set of sites upon 6 or 24 hours of MS-275 treatment or inactivation/deletion of HDAC1/2/3. Acetyl(K)sites are classified as HDAC1/2, HDAC3 or HDAC1/2/3 preferential substrates. (A) Histone substrates. For simplicity, individual hits from histone sub-variants are summarized according to their main types (indicated on the right). Positions of acetyl-lysines in the histone protein are indicated on the left. N-terminal methionines are not considered, to fit the commonly used histone site code. (B) Non-histone substrates, grouped according to their biological function. Corresponding gene names and positions of acetyl-lysines are indicated on the left. *, **: The information for quantification of this site comes from a *dual or **triple acetylated peptide where only one site was confidentially allocated.

Inactivation of individual HDACs reflects to large extent the acetylation profiles of pharmacological inactivation (**S1 Table** and **Fig 4**). Acetylation sites increased upon HDAC1 or HDAC2 inactivation were highly similar to each other and particularly resembled profiles of 6 hour MS-275 treated cells (82% overlap of 6 hour MS-275 treated cells to HDAC1/2 CI cell lines). In contrast, acetylation sites increased by HDAC3 inactivation differed from those affected by HDAC1/2 inactivation and were less responsive to pharmacological HDAC inhibition (32% overlap of 6 hour MS-275 treated cells to HDAC3 CI cell lines). Among the HDAC cell lines, KO HDAC3 CI cells displayed the largest number of significantly hyperacetylated proteins (175), followed by HDAC3 KO cells (104) and only a minor fraction was found to be hyperacetylated in WT HDAC3 CI cells (38). Importantly, we identified confident HDAC1/2- and HDAC3-specific histone and non-histone substrates, which were independent of the level of HDAC inactivation: 15 proteins with increased acetylation sites were overlapping in both HDAC1 CI clones, while 19 and 25 targets were identified in both HDAC2 CI clones and HDAC3 CI clones, respectively. Of the 25 overlapping targets of HDAC3 CI clones, 20 also displayed an overlap with the HDAC3 KO.

Next we specifically analyzed effects of HDAC1/2/3 inactivation on histone acetylation (**Fig 4A**). We determined acetylation sites which were elevated upon expression of HDAC1/2 CI mutants (**upper panel**), inactivation or deletion of HDAC3 (middle panel), or which were regulated by all three class I deacetylases (**lower panel**). The selected sites were also found hyperacetylated upon MS-275 treatment. HDAC1/2 inactivation evoked stronger effects on histone acetylation sites compared to HDAC3 CI or KO clones.

Multiple detected lysine acetylation sites on histones H1, H2A, H2B type 1/2 as well as K23 on histone H3, were preferentially regulated by HDAC1/2 deacetylase function. HDAC3 inactivation caused a higher tendency towards increased K12, K15 and K16 acetylation on histone H2B type 3-B. Notably, HDAC1, HDAC2 and HDAC3 inactivation, as well as deletion of HDAC3 increased H3 and H4 histone acetylation sites, such as H3 K9ac, H3 K14ac, H4 K8ac and H4 K16ac which have well-known functions in transcriptional regulation. We also noticed that observed effects were stronger if a particular site was also covered as multi-acetylated peptide, confirming the frequently observed histone mark co-occurrence and dependency [89].

To expand our findings on histone acetylation and to additionally get insight into the dynamics, we performed a targeted proteomics approach based on PRM, using cells that were treated for 2–24 hours with MS-275 (**S3A Fig, right panel,** and **S2 Table**). 2 hours of MS-275 treatment already caused a slight, but significant increase of few acetylation sites such as K9ac and K14ac on histone H3, and K5ac, K8ac, K12ac and K16ac on histone H4. Following a gradual increase after 6 hours of treatment, some acetylated peptides were elevated 20-90-fold at the 24 hours time point. We observed again that the majority of detected peptides were multi-acetylated. MS-275 responsive acetylation sites identified by the targeted approach (**S3A Fig, right panel,** and **S2 Table**) were in accordance with the results from the initial shotgun experiment (summarized in **S3A Fig, left panel** and listed in **S1 Table**).

We further examined changes in acetylation patterns of non-histone proteins (**Fig 4B** and **S1 Table**). We determined the top deacetylation targets of HDAC1/2 (**upper panel**) and HDAC3 (**lower panel**) and grouped them according to their biological functions.

Upon inactivation of HDAC1 or HDAC2, we identified hyperacetylated sites on multiple components of HDAC1/2 associated co-repressor complexes, such as MTA2 and CHD4 of the NURD complex, COREST (RCOR1) and RCOR3 of the COREST complex, ELMSAN1 of the MIDAC complex and RERE, which acts as scaffold for several HDAC complexes [13, 90]. Acetylation of COREST (RCOR1), a protein with a single responsive acetylation site (K14), was also found increased upon MS-275 treatment by IP-Western blot analysis (**S3B Fig**).

Interestingly, our global acetylation analysis further revealed that one particular acetylation site (K412) within the HDAC1 enzyme itself was elevated upon MS-275 treatment and upon expression of inactive HDAC1, but not HDAC2 or HDAC3 (**Fig 4B, upper panel,** and **S1 Table**). In addition, acetylated lysine residues within components of HAT (histone acetyltransferase) complexes, such as MEAF6, the bromodomain containing reader proteins BRPF1, BRPF3, and HAT enzymes (KAT6A, CREBBP) [91] were preferentially increased upon expression of inactive HDAC1/2. Similarly, we determined elevated acetylation sites on multiple other chromatin-associated proteins, such as transcriptional regulators and transcription factors, including zinc finger containing proteins. Notably, many of the acetylation sites preferentially regulated by HDAC1/2 activity were also increased upon MS-275 treatment.

Upon inactivation and/or deletion of HDAC3 (**Fig 4B, lower panel,** and **S1 Table**), we identified elevated acetylation sites on components of HDAC3-associated NCOR/SMRT nuclear receptor corepressor complexes, including NCOR1, NCOR2 and TBL1XR1 [92]. HDAC3 deacetylates additional proteins associated with nuclear receptor binding including ACTN4, JMJD1C, ARID1A [93–95], or the proto-oncogene SKI [96]. Interestingly, these proteins, in addition to other identified HDAC3 targets including MB21D2 and the melanoma-associated antigen MAGED1, also have cancer-associated functions [95,97–100]. Inactivation of HDAC3 also resulted in increased acetylation sites on the RNA-binding proteins ZC3H7B and SAMHD [101,102].

In summary, histone acetylation sites are regulated to a larger extent by HDAC1/2 catalytic activity, together with sites found on chromatin-associated factors. In contrast, HDAC3 seems to have a trend towards deacetylation of non-histone targets with more diverse cellular functions.

### Distinct effects of catalytic inactivation of HDAC1/2 and HDAC3 on gene expression

**Fig 5:**
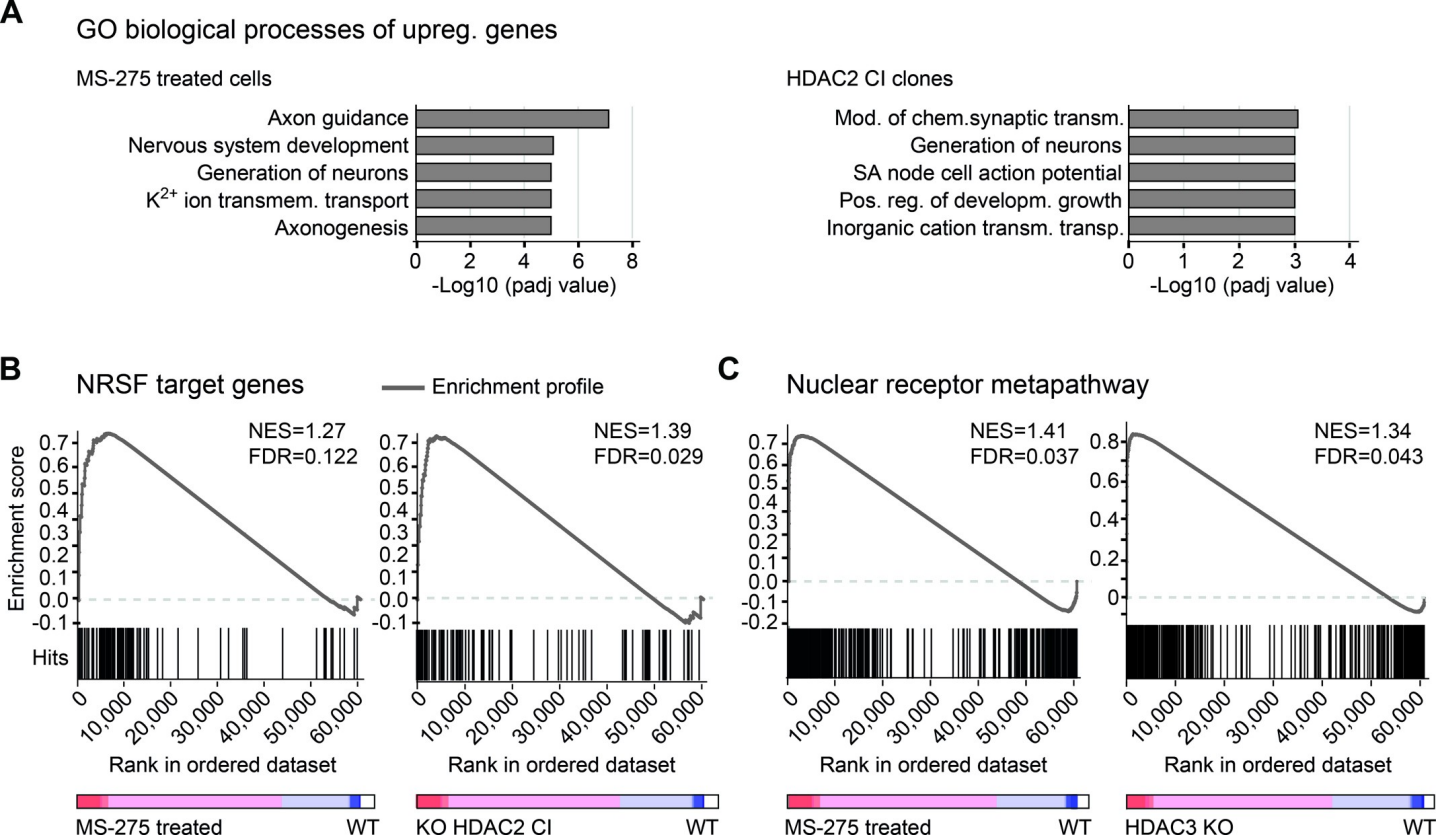
Transcriptional control by HDAC1/2/3 catalytic activity. (A) Enriched gene ontology terms of significantly upregulated genes upon MS-275 treatment (left panel) or HDAC2 inactivation (right panel) (≥ 2-fold change over wildtype cells, padj-value ≤ 0.05), determined with the Enrichr tool. Only genes elevated in both HDAC2 CI clones (wildtype and knockout background) were considered for analysis. (B) Gene set enrichment analysis (GSEA) showing the correlation of NRSF target genes with transcription profiles of MS-275 treated cells (left) and KO HDAC2 CI cells (right). (C) GSEA of genes associated with the nuclear receptor metapathway of transcriptome data of MS-275 treated cells (left) and HDAC3 KO cells (right). (B-C) Running enrichment score (grey line), reflecting to which degree NRSF or nuclear receptor metapathway regulated genes from defined, publicly available lists are overrepresented at the extremes of ranked gene expression data from the indicated cell lines. The gene tags (“Hits”, black lines) indicate the location of the genes from the defined lists within the ranked datasets. The colored bar shows positive (red) and negative (blue) correlations with indicated cell lines. NES (normalized enrichment score) and FDR q-value are indicated for each analysis.

Increased acetylation sites on histones and chromatin-modifying enzymes might impact the transcriptional landscapes of the individual HDAC1/2/3 cell lines. Therefore, we performed RNA-seq analysis (**Figs 5 and S4, and S3 Table**). To compare changes in gene expression resulting from genetic inactivation to effects caused by pharmacological inactivation, we analyzed cells with partially of fully inactivated HDAC1/2/3 enzymes and cells treated with the HDACis JQ12 or MS-275. Due to the strong effects caused by HDAC3 deletion in previous parts of the study, we again included HDAC3 KO cells. A sample distance heatmap based on the Euclidean distance (**S4A Fig**) and a principal component analysis (**S4B Fig**) show that the three biological replicates for each cell line or treatment were in good agreement with each other, confirming the technical quality of the data set. HDAC3 cell lines, including WT HDAC3 CI, KO HDAC3 CI and HDAC3 KO cells, are highly similar to each other. They differ considerably from cells expressing inactive HDAC1/2 transgenes, indicating distinct functions in gene expression regulation. Cell lines expressing HDAC1/2 CI enzymes in KO as well as in WT background each form neighboring clusters (**S4A Fig**). This confirms the high degree of overlapping functions of HDAC1 and HDAC2 enzymes as orthologous genetic alterations cause similar effects on gene expression. In addition, cells treated with the HDACis MS-275 and JQ12 are more similar to HDAC1/2 cell lines than to HDAC3 cell lines. The high similarity of HDAC1/2 CI expressing cells to each other and their distinction from cells with inactivated and/or ablated HDAC3 enzyme resembles the findings of the acetylome study.

To get an overview of altered biological processes upon HDAC1/2/3 inactivation, we performed gene ontology (GO) term enrichment analysis of significantly upregulated genes (≥ 2-fold changes over wildtype cells, padj-value ≤ 0.05). Thereby, pharmacological HDAC inactivation by MS-275 and JQ12 particularly increased the expression of genes associated with the nervous system (**Figs 5A, left panel,** and **S4C, upper panel, respectively**). Next, we assessed the GO enrichment for upregulated genes upon expression of the inactive HDAC enzymes. Therefore, we focused on the intersection of genes significantly increased upon both partial and full inactivation. In particular, upon expression of inactive HDAC2, and also HDAC1, we found GO terms related to neuronal functions enriched (**Figs 5A, right panel** and **S4C, lower panel**), similar to observations for HDACi-treated cells.

Notably, our acetylome analysis has revealed that members of individual HDAC1/2 complexes, such as the COREST complex components COREST (RCOR1) and its less well characterized paralogue RCOR3 [103], displayed increased acetylation sites upon MS-275 treatment and inactivation of HDAC1/2 (**Fig 4B, upper panel**). Interestingly, the COREST complex is known for its repressive function on neuronal-specific genes in non-neuronal cells, mediated *via* specific binding through the associated transcription factor REST [104,105]. Gene Set Enrichment Analysis (GSEA) shows that REST target genes are overrepresented in the genes upregulated upon HDAC2 inactivation or MS-275 treatment (**Fig 5B)**. Other HDAC1/2 CI cell lines showed a similar effect using the same analysis (WT HDAC2 CI: normalized enrichment score (NES) = 1.25, FDR = 0.111; WT HDAC1 CI: NES = 1.23, FDR = 0.164; KO HDAC1 CI: NES = 1.10, FDR = 0.267). In line with this, we also observed a significant decrease of COREST-associated deacetylase activity upon expression of inactive HDAC1/2 (**S5A and S5B Fig**).

Given that HDAC3 associated complexes function in nuclear receptor-mediated transcriptional repression [106], and that inactivation of HDAC3 caused hyperacetylation of nuclear receptor-associated proteins (**Fig 4B, lower panel**), we next investigated whether genes regulated by nuclear receptors are enriched upon HDAC3 deletion/inactivation. Expression profiles of HDAC3 KO cells and cells treated with MS-275 displayed a significant enrichment of genes involved in the nuclear receptor metapathway (**Fig 5C**) and expression of inactive HDAC3 caused similar effects (WT HDAC3 CI: NES = 1.17, FDR = 0.144; KO HDAC3 CI: NES = 1.25, FDR = 0.082).

In summary, these findings suggest that changes in gene expression profiles caused by HDAC1/2 and HDAC3 inactivation/deletion are connected with the annotated function of their non-histone deacetylation targets.

### Genetic and pharmacological HDAC inactivation sensitizes cells for tumor drugs

**Fig 6:**
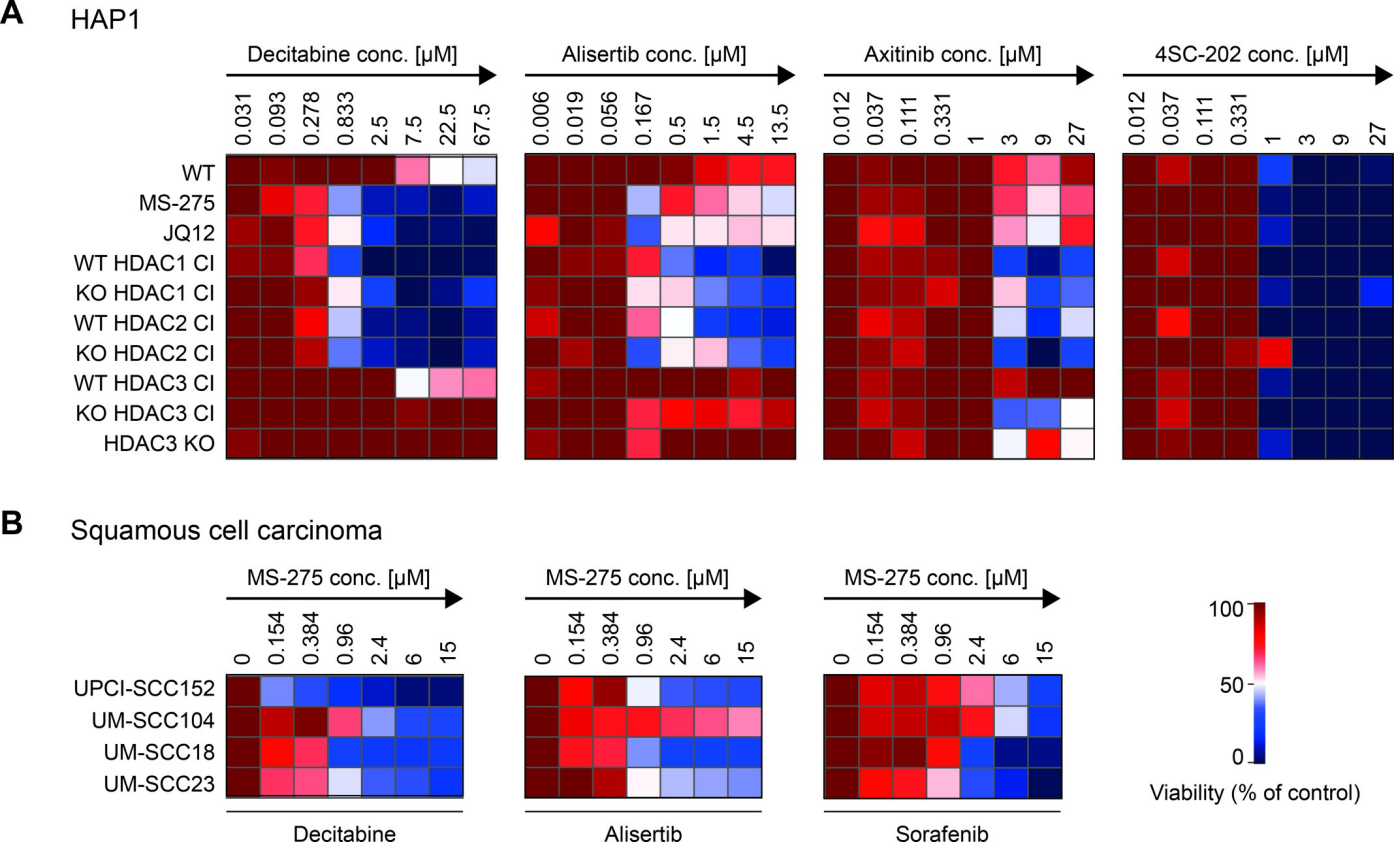
HDAC inactivation sensitizes cells to clinically approved anti-tumor drugs. (A) The heatmaps illustrate the sensitivity of different HAP1 HDAC1/2/3 CI cell lines or HDACi treated cells to anti-tumor drugs. JQ12 or MS-275 treated cells, HDAC1/2/3 CI expressing cells (in wildtype and knockout background) and HDAC3 KO cells were incubated with increasing concentrations of following drugs: decitabine, alisertib, axitinib and 4SC-202 (negative control), indicated from left to right panel. The screen was performed in duplicate. Mean values, normalized to plate internal positive and negative controls, are shown as relative viability (in %) over the controls. (B) Combinatorial effects of MS-275 and anti-tumor drugs determined for squamous cell carcinoma cell lines. Following cell lines were included: UPCI-SCC152, UM-SCC104, UM-SCC18 and UM-SCC23. The IC50 for the respective tumor drug was determined for each cell line. The heatmaps show how relative IC50 values change upon simultaneous treatment with MS-275 (concentrations are indicated). The color bar represents the relative viability of duplicate screens (in %) over the control. The control was treated with the indicated tumor drug, but not with MS-275 (set as 100%).

Global acetylome and transcriptome analysis suggest that genetic inactivation of individual HDACs effectively mimics pharmacological inactivation. Based on the fact that HDACis can have synergistic effects with tumor drugs [49], we tested if inactivation/deletion of particular HDACs causes sensitizing effects. We used a collection of clinically approved cancer drugs that have been previously evaluated for *ex vivo* chemosensitivities in tumor biopsies in the context of the clinical trial EXACT (EXtended Analysis for Cancer Treatment) [58,107]. Based on a pilot screen, we selected three compounds, the DNMT inhibitor decitabine, the Aurora A inhibitor alisertib and the tyrosine kinase inhibitor axitinib for more detailed investigation. In a follow-up screen, decitabine, alisertib and axitinib were used in an 8-point dose-response assay (**Fig 6A**). The HDACi 4SC-202, which showed no effect in the pilot experiment, was included as control. We included HAP1 wildtype cells, next to cell lines with partial of full inactivation of HDAC1/2/3 and cells pre-treated with MS-275 or JQ12 before addition of the tumor drugs. Following 72 hours of treatment with the different doses, the viability of the cells was determined. Pre-treatment with the HDACis or inactivation of HDAC1 or HDAC2 sensitized HAP1 cells to decitabine and similar trends were observed for alisertib and axitinib (**Fig 6A**). Cells expressing inactive HDAC3 in the KO background, as well as HDAC3 KO cells were slightly more sensitive towards axitinib. None of the transgenic HDACs or treatments sensitized the cells to the negative control 4SC-202.

To extend our analysis to more relevant tumor cell lines, we examined the sensitizing effect of class I HDAC inactivation in HNSCC (head and neck squamous cell carcinoma) cell lines. We used the human papillomavirus (HPV)-positive head and neck cancer cell lines UPCI-SSC152 and UM-SCC104 [108], in addition to the HPV-negative head and neck oropharyngeal and laryngeal cancer cell lines UM-SCC18 and UM-SCC23. Since some of the cell lines were not responsive to axitinib, we used sorafenib instead, another tyrosine kinase inhibitor with the same molecular target (VEGFR). First, we determined the IC50 value for each of the four tumor drugs in the different cell lines. Next, we monitored how increasing concentrations of MS-275 change these IC50 values (**Fig 6B**). Class I HDAC inhibition sensitized all cell lines to the three tumor drugs, although the effect on UM-SCC104 was mild. This cell line with episomal HPV 16 expresses high levels of the viral oncoprotein E7 [108], which might result in reduced effectiveness of MS-275. Of note, synergistic effects of HDACis with decitabine, alisertib, sorafenib and axitinib have been previously demonstrated [109–112] and these combinations are currently being tested in clinical trials [48,113,114]. In summary, our novel cell models are well suited for examining isoform-specific HDAC inactivation on cellular context.

## Discussion

Herein, we describe consequences of loss-of-activity mutations of the class I HDACs HDAC1, HDAC2 and HDAC3 in comparison with class I HDAC inhibitors. Our detailed approach suggests that the expression of particular catalytically inactive mutants reflects inhibition of the enzyme, allowing to identify isoform-dependent catalytic functions.

HDAC inhibitor treatment affects tumor cell viability by induction of cell death and inhibition of proliferation [47]. Inactivation of either HDAC1 or HDAC2 causes only minor effects on cell viability, whereas loss of HDAC3 or its activity led to DNA damage and apoptosis resulting in severely reduced cell viability. In agreement with these data HDAC3 deletion in different cell types inhibits proliferation or induces genomic instability and cell death [39,40,82]. In contrast, combined loss of HDAC1 and HDAC2 strongly affects cell viability, while deletion of only one of the two enzymes has little impact [8,11,38,44,115–118].

In addition to cell-based assays, we integrated multi-OMICS approaches to determine effects of HDAC1/2/3 inactivation. We examined differences in cellular acetylomes upon treatment with MS-275 or expression of particular inactive HDAC1/2/3 isoforms (**Figs 3 and 4**). The catalytic activity of all three enzymes seems essential for deacetylating histone sites, with HDAC1/2 exerting the strongest effects. This is in agreement with the higher associated deacetylase activity of precipitated HDAC1/2 towards acetylated histones compared to HDAC3 enzyme (**S1A and S1C Fig**), negative effects of inactive HDAC2 on total cellular histone deacetylase activity (**Fig 2B**) and significant effects of HDAC1/2 deletion on total cellular activity [20,38]. Inactivation of HDAC1, HDAC2 or HDAC3 increased acetylation on well-known sites of histones H3 and H4 (**Fig 4A**). Interestingly, many of the preferred sites of HDAC1/2 correspond to linker histone H1 and variants of histones H2A and H2B. Increased acetylation of these sites was also observed upon pharmacological HDAC inhibition in our study and previous studies [85]. While H2A and H2B acetylation sites are less well characterized compared to sites on H3 and H4 histones, their importance in regulating transcription, especially by their N-terminal domain, was demonstrated in previous studies [119,120]. Furthermore, some of these sites might be deregulated or play a role in cancer and autoimmune diseases [121,122].

Importantly, we unraveled multiple non-histone deacetylation substrates of individual HDAC1/2/3 enzymes (**Fig 4B**). HDAC1/2 catalytic inactivation resulted in increased acetylation sites of numerous components of HDAC1/2 associated co-repressor complexes, reader proteins and transcription factors. Notably, many of these chromatin associated factors were already hyperacetylated upon 6 hours of MS-275 treatment, suggesting that acetylation of proteins closely interacting with HDACs is highly dynamic.

In this context it is remarkable that multiple histone acetyltransferases (HATs) and HDAC1 itself are potential HDAC1/2 targets, suggesting a cross-talk between these antagonistic enzymes. We detected multiple increased acetylation sites within the CREBBP (CBP) protein upon HDAC1/2 inactivation or MS-275 treatment. Interestingly, the corresponding sites are part of an autoregulatory loop of the enzyme [123,124]. According to previous reports, hyperacetylation of this region leads to increased histone acetyltransferase (HAT) activity [123,125–127]. Pharmacological HDAC inhibition also led to hyperacetylation of corresponding sites within the CBP homolog p300 [5]. In addition, HAT-associated reader proteins (BRPF1, BRPF3) [128] showed increased acetylation sites upon HDAC inhibitor treatment (this study **Fig 4B** and [85]), and genetic HDAC1 or HDAC2 inactivation. On the other hand, p300 was shown to acetylate HDAC1 at K412 [129] and this acetylation mark is sensitive to HDAC1 inactivation (**Fig 4B**). This suggests that specific class I HDACs might affect the cellular function of HATs and *vice versa*.

Upon inactivation or deletion of HDAC3, we detected hyperacetylated sites on components of NCOR/SMRT complexes [130,131], in addition to other proteins associated with nuclear receptor binding (**Fig 4B**). For example, ARID1A, a subunit of the SWI/SNF chromatin remodeling complex, as well as the H3K9 demethylase JMJD1C have been identified as interactors of nuclear hormone receptors [93–95]. Both of them, in addition to another HDAC3 deacetylation substrate, the actin-binding protein ACTN4, comprise the LXXLL motif, which is known to facilitate interaction with nuclear receptors [95,132,133]. ACTN4 is furthermore suggested to negatively regulate the activity of the class IIa deacetylase HDAC7, which functions together with HDAC3 in NCOR/SMRT complexes [18,134,135]. The additional involvement of class II enzymes in NCOR/SMRT/HDAC3 complexes might explain why some potential HDAC3 substrates identified in our acetylome study, did not show increased acetylation sites in MS-275 treated cells.

The proto-oncogene SKI, by interactions with HDAC3 containing NCOR/SMRT complexes, is also involved in the transcriptional regulation *via* several nuclear receptors including the retinoic acid receptor and thyroid hormone receptor [96,136–138]. Interestingly, in addition to SKI, many other putative HDAC3 deacetylation substrates have cancer related functions [95,97–100]. Notably, the melanoma antigen MAGED1 [139], which also displays hyperacetylated sites upon HDAC3 deletion/inactivation, plays a major role in apoptosis and cell cycle arrest [140,141], which could provide a further link between HDAC3 function and the reduced viability of HDAC3 CI or HDAC3 KO clones. Besides, potential HDAC3 deacetylation targets also involved few RNA-binding proteins, including for example ZC3H7B, which plays a role during virus infection [101,102].

Altogether, our acetylome analysis revealed novel HDAC1/2/3 deacetylation substrates that differ in their cellular function. HDAC3 cellular targets were more diverse while HDAC1/2 inactivation primarily induced hyperacetylation on histones and chromatin-associated non-histone substrates. Importantly, many increased acetylation sites detected upon HDAC1/2 inactivation were also hyperacetylated upon MS-275 treatment, suggesting that HDAC1/2 CI mutants effectively mimic pharmacological HDAC inhibition.

Our transcriptome analysis (**Figs 5 and S4**) furthermore revealed that gene sets related to similar biological processes are derepressed by both HDAC inhibitor treatment and by genetic HDAC inactivation. Similar to the acetylome analysis, different processes were affected by HDAC1/2 and HDAC3 inactivation suggesting a division of labor among class I HDACs. Importantly, gene sets that were overrepresented in the individual HDAC cell lines are regulated by factors, which were identified as hyperacetylated upon HDAC1/2 or HDAC3 inactivation. Future experiments will be required to evaluate whether the reversible acetylation of these factors has functional consequences. RCOR1 (COREST) and also its homologue RCOR3, were among the top targets of HDAC1/2 deacetylase activity (**Fig 4B**) and are components of the HDAC1/2 associated COREST complex, a well-known transcriptional regulator of neuronal genes [103,142]. Genes upregulated by MS-275 or JQ12 treatment or HDAC1/2 genetic inactivation showed an enrichment of GO terms associated with neuronal function (**Figs 5A and S4C**). The derepression of neuronal genes upon pharmacological HDAC inhibition is consistent with previous work [76]. In addition, it is known that HDAC2 plays important roles in the control of brain development and learning [21,46,143–145]. In line with a reduction of COREST-associated activity upon incorporation of inactive HDAC1/2 (**S5A and S5B Fig**), REST target genes were enriched in expression profiles of HDAC1/2 CI expressing cells and MS-275 treated cells (**Fig 5B**). Notably, the strongest effects were observed upon inactivation of HDAC2, which is in accordance with the previously reported importance of HDAC2 for function of the COREST complex [143]. This effect, caused by the use of catalytically inactive HDAC1/2 mutants, might also be interesting in respect to the promising development of complex-specific HDAC inhibitors [13,146], including recently developed hybrid LSD1/HDAC inhibitors specifically targeting the COREST complex [147].

Our acetylome analysis revealed a strong link between HDAC3 and annotated nuclear receptor binding-associated proteins. Upon inactivation/deletion of HDAC3, NCOR1 and NCOR2, components of HDAC3 associated NCOR/SMRT complexes, displayed the highest increase of acetylated sites (**Fig 4B**). Importantly, they function by tethering HDAC3 to transcriptional repressors including unliganded nuclear receptors [106], such as the thyroid hormone receptor [148–150] retinoic acid receptor [17,151] and vitamin D receptor [152]. In line with this, gene expression profiles of cells with deleted/inactivated HDAC3, in addition to MS-275 treated cells, showed an enrichment of genes functioning in nuclear receptor pathways (**Fig 5C**).

Finally, we showed that our novel HDAC model is suitable for chemical screens (**Fig 6**). Using a panel of clinically approved cancer drugs we tested whether the inactivation or deletion of individual HDAC enzymes can sensitize cells to these drugs (**Fig 6A**). We identified the DNMT inhibitor decitabine, the Aurora A kinase inhibitor alisertib and the tyrosine kinase inhibitor axitinib as synergistic hits for class I HDACs. In all cases, the expression of either inactive HDAC1 or HDAC2 was sufficient to sensitize cells for tumor drug treatment. In contrast, the inactivation of HDAC3 showed no consistent effects. In addition, especially in case of decitabine treatment, HAP1 cells were also sensitized upon treatment with JQ12 or MS-275. These findings were confirmed in several HNSCC tumor cell lines (**Fig 6B**). The DNMT inhibitor decitabine is an FDA-approved effective chemotherapeutic agent for acute myelogenous leukaemia with anti-cancer effects also in head and neck cancer cells [153]. Alisertib is an investigational Aurora kinase A-specific inhibitor, effective in head and neck cancer cells [154]. Axitinib is among the VEGF inhibitors, which are being tested in various clinical trials of head and neck squamous cell carcinoma (reviewed in [155]). Of note, synergisms between HDAC inhibitors and decitabine, alisertib, sorafenib and axitinib have been previously demonstrated [109–112,156–158]) and treatment with these drugs in combination with HDACis is currently being tested in clinical trials [48,113,114].

Throughout our comprehensive analyses, results obtained by inactivation of HDAC1 and HDAC2, and to a lesser extent HDAC3, overlap with those obtained by HDAC inhibitor treatment. Of note, the observed effects for the inactivation of each of the analyzed HDACs was very similar in wildtype and KO background and inactivation of HDAC1 and HDAC2 caused overlapping effects. These results are compatible with the idea that these mutants mimic isoform-specific pharmacological inhibition. Consistent with these data, expression of inactive HDAC1 or HDAC2 evoked an isoform-specific effect on mouse development [46]. Due to their partial redundancy, genetic knockout/knockdown screens might fail to detect important but overlapping cellular functions of HDAC1 and HDAC2. In contrast to the HDAC1/HDAC2 KO, expression of these dominant negative mutants might prevent the compensation by the respective paralog.

In line with a unique cellular function, deletion of HDAC3 has a strong effect on cell viability, the acetylome and the transcriptome. Mouse studies have identified an activity-independent function of HDAC3 in transcriptional regulation in the liver [42] and during spermatogenesis [159] suggesting a structural function of the enzyme. On the other hand, HDAC3 activity was shown to be required for B-cell development [160]. Taken together, we have successfully generated a genetic toolbox to dissect catalytic functions of the class I deacetylases HDAC1/2/3 and provide extensive acetylproteomic and transcriptomic resources for future biological analyses. Our data show that the genetic inactivation of individual class I HDACs effectively mimics isoform specific inhibitors. For future studies, the set-up of inducible systems will allow the fast inactivation of individual class I HDACs. Mouse models with cell type-specific replacement of wildtype HDACs by catalytically inactive mutants will further enable to study enzymatic HDAC functions in development and disease in a complex organism.

## Supporting information

S1 Fig Deacetylase activity of class I HDAC enzymes. (A) Immunoprecipitation analysis of endogenous HDAC1, HDAC2, HDAC3 and HDAC8 enzymes from cellular extracts of HAP1 wildtype (WT) cells. Cells with a knockout (KO) of the respective HDACs were used as negative controls. Immunoprecipitates were examined by Western blot analysis using specific antibodies against the enzymes (upper panel) and HDAC activity assays (lower panel). (B) Illustration of the targeting strategy to establish transgenic HDAC1/2/3 CI cell lines. HDAC transgenes (red) provided by the pVJV vector, were integrated into the AAVS1 locus within the HAP1 genome (blue) via CRISPR/Cas9 technology and homologous recombination (LHA…left homology arm, RHA…right homology arm). Transgenes were expressed under control of the EF-1α promoter (brown). (C) Pair-wise immunoprecipitation of comparable amounts of FLAG-tagged HDAC1/2/3 WT and CI enzymes to assess their deacetylase activity. FLAG immunoprecipitates were analyzed on Western blots (upper panel) using the FLAG antibody. Precipitates of wildtype HAP1 cells are shown as negative control. Associated deacetylase activities of HDAC WT and CI enzymes are presented below. (A+C) Bar graphs represent mean values ± standard deviation (SD) of 4 (A) or 3 (C) biological replicates and the significance was determined by Welch‘s t-test. *p < 0.05, **p < 0.01, ***p < 0.001.
(TIF)

S2 Fig Consequences of HDAC1/2/3 inactivation on apoptosis and DNA damage. (A) Bar graph representing the percentage of apoptotic cells of cultured cell lines, including wildtype cells, HDAC1/2/3 CI expressing cells (wildtype and knockout background) and HDAC3 KO cells. Apoptotic cells were determined by flow cytometry based on signals for cleaved caspase 3. (B) Representative immunofluorescence images of wildtype control cells and cells with inactivated/deleted HDAC3. DNA damage was analyzed with an antibody specific for γ-H2AX and DNA was stained with Hoechst dye. (C) Quantification of γ-H2AX foci determined by immunofluorescence analysis from WT HDAC1/2/3 CI cells (left panel), KO HDAC1/2/3 CI cells (middle panel) and HDAC3 KO cells (right panel) next to wildtype cells as control. Mean values ±SD of 3 (A) or 4–6 (C) biological replicates are shown. Significance was determined by one-way ANOVA. The pairwise comparison of HDAC3 KO cells to wildtype cells (in (C), right panel) was performed using Welch‘s t-test. **p < 0.01, ***p < 0.001.
(TIF)

S3 Fig Increase of lysine acetylation sites following HDAC inhibition. (A) Dynamics of histone acetylation. A selected set of histone acetyl(K) sites, based on findings from the MS-shotgun experiment (left panel), was further investigated by targeted proteomics to monitor changes after 2, 6 and 24 hours of MS-275 treatment (right panel, n = 4). Increased acetylation is shown as log2 fold change over untreated wildtype cells. Individual hits from histone sub-variants are summarized according to their main types (indicated on the right). Positions of acetyl-lysines in the histone protein are indicated on the left. N-terminal methionines are not considered, to fit the commonly used histone site code. *, **: The information for quantification of this site comes from a *dual or ** triple acetylated peptide where only one site was confidentially allocated (for MS shotgun data only). (B) Assessment of COREST (RCOR1) acetylation by acetyl(K)-IP and subsequent Western blot analysis using untreated or 24 hour MS-275 treated cells. The blot was incubated with indicated antibodies (on the left). Vinculin was used as loading control. n = 2
(TIF)

S4 Fig Analysis of the transcriptome. (A) Sample distance heatmap of RNA seq replicas from HDAC1/2/3 CI expressing cell lines (wildtype and knockout background), MS-275 or JQ12 treated cells (24 hours) and wildtype cells. The color code is based on the Euclidean distance of read counts after variance stabilizing transformation, while the dendrogram represents a hierarchical cluster analysis with the complete linkage method. (B) Two-dimensional principal component analysis (PCA) of RNA-seq transcriptome profiles of wildtype, MS-275 or JQ12 treated cells, HDAC1/2/3 CI expressing cells (wildtype and knockout background) and HDAC3 KO cells. (C) Enriched gene ontology terms of significantly upregulated genes by JQ12 treatment (upper panel) and HDAC1 inactivation (lower panel) (≥ 2-fold change over wildtype cells, padj-value ≤ 0.05), determined with the Enrichr tool. Only genes elevated in both HDAC1 CI clones (wildtype and knockout background) were considered for analysis.
(TIF)

S5 Fig Analysis of the HDAC1/2 associated regulator COREST. (A-B) Analysis of COREST (RCOR1) associated HDAC activity upon incorporation of inactive HDAC1 or HDAC2 enzymes. COREST was immunoprecipitated from extracts of (A) wildtype, WT HDAC1 CI and KO HDAC1 CI cells and of (B) wildtype, WT HDAC2 CI and KO HDAC2 CI cells. Inputs (left panels) and immunoprecipitates (middle panels) were analyzed by Western blotting using antibodies indicated on the left. Bar graphs (right panels) represent changes of COREST associated deacetylase activity, measured by incubating the precipitates with acetylated histones. Mean values ± SD of 3 biological replicates are shown and significance was determined by Welch‘s t-test. *p < 0.05, **p < 0.01, ***p < 0.001.
(TIF)

S1 Table Summary of quantified acetyl(K) sites over MS shotgun experiments of our study (15 experimental conditions, three replicates, analyzed in three TMTpro 16plex setups in total). The table includes results from the differential expression (DE) testing analysis of protein-normalized acetyl(K)sites (columns C-AL), protein-normalized TMTpro reporter ion intensities of quantified acetyl(K) sites in different conditions (columns AM-BP, and hidden columns BQ-CH), as well as results from DE-testing of proteinGroups (hidden columns CI-DI), and selected columns from the MQ output tables acetyl(K)sites.txt (hidden columns DJ-EB). To generate the table the output from R-script "acetylKSites_analysis_prot_norm" (**S1 Material**) has been processed using MS-Excel. The table covers information on MS-275 treated wildtype cells, HDAC1-3 catalytic inactive mutants expressed in wildtype and corresponding KOs, and HDAC3KO cells. Results obtained from additional experimental conditions covering HDAC1KO and HDAC2KO cells as well as different HDAC8-related strains are hidden columns BQ-CH).
(XLSB)

S2 Table Summary of quantified acetyl(K) sites identified by our targeted proteomics approach (4 experimental conditions, four replicates). This table includes Differential Expression (DE) Testing analysis of acetylated peptides of histones (normalized to unmodified peptides) (columns D-F), corresponding statistical analysis (G-J), summed fragment ion intensities (K-Z) and additional information (AA-AB). The table covers information on MS-275 treated wildtype cells (2, 6 or 24 hour time points).
(XLSB)

S3 Table Summary of differential gene expression analysis. The different worksheets show results obtained by DESeq2 analysis for JQ12 or MS-275 treated wildtype cells, HDAC1-3 catalytic inactive mutants expressed in wildtype and corresponding KOs, and HDAC3 KO cells. Relative expression was calculated relative to wildtype cells. RNA seq experiments were performed using biological triplicates.
(XLSB)

S1 Material R scripts used for proteomics data analysis and reports in html-format summarizing the analysis and quality control. (ZIP)

S1 Data Numerical data of the graphs. (XLSX)
