# Supplementary figures and images for: A toolbox for class I HDACs reveals isoform specific roles in gene regulation and protein acetylation
Source: PLoS Genet. 2022 Aug 22;18(8):e1010376. doi: 10.1371/journal.pgen.1010376 (PMC9436093; doi:10.1371/journal.pgen.1010376)

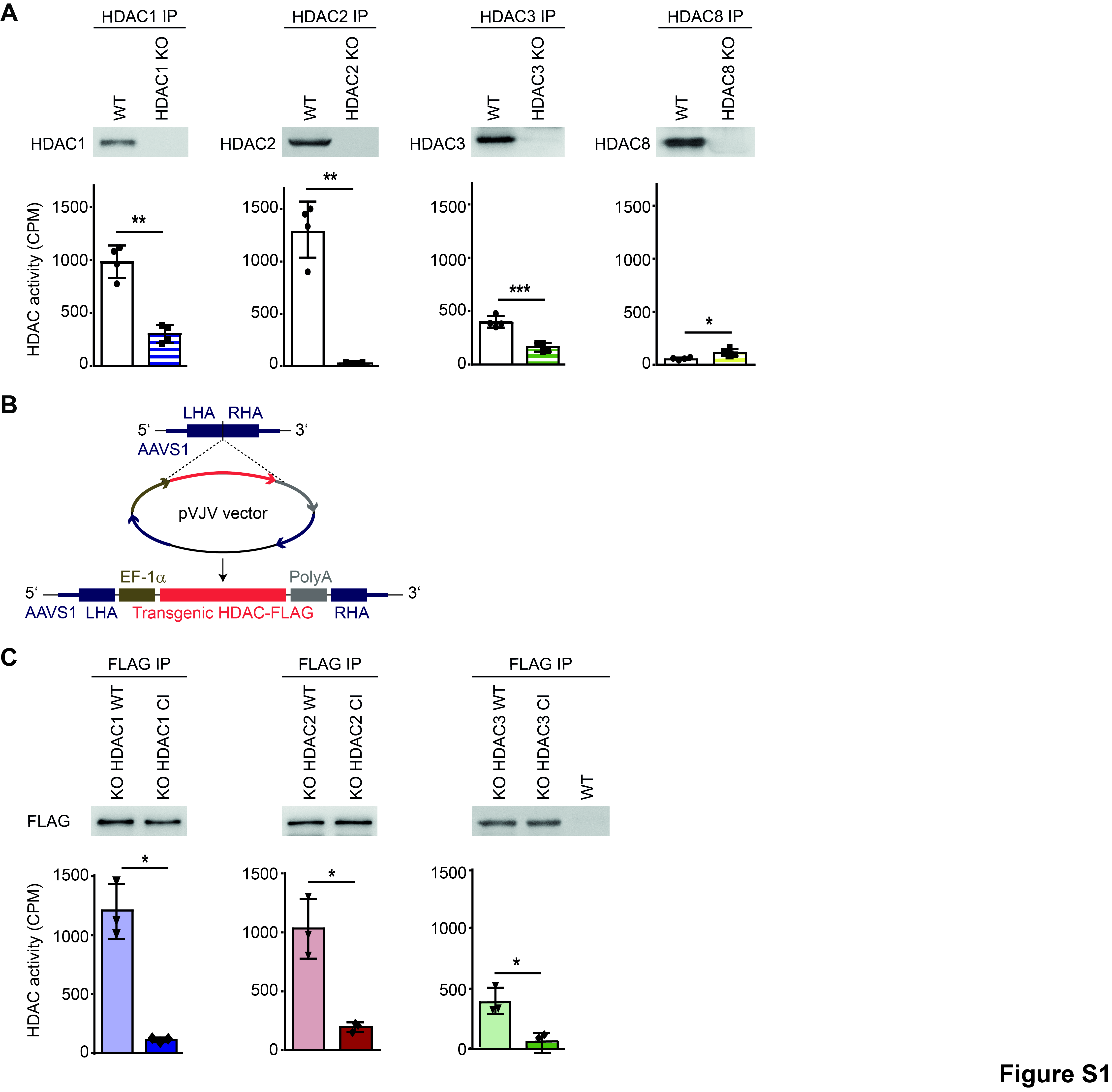

Supplement: S1 Fig — (A) Immunoprecipitation analysis of endogenous HDAC1, HDAC2, HDAC3 and HDAC8 enzymes from cellular extracts of HAP1 wildtype (WT) cells. Cells with a knockout (KO) of the respective HDACs were used as negative controls. Immunoprecipitates were examined by Western blot analysis using specific antibodies against the enzymes (upper panel) and HDAC activity assays (lower panel). (B) Illustration of the targeting strategy to establish transgenic HDAC1/2/3 CI cell lines. HDAC transgenes (red) provided by the pVJV vector, were integrated into the AAVS1 locus within the HAP1 genome (blue) via CRISPR/Cas9 technology and homologous recombination (LHA…left homology arm, RHA…right homology arm). Transgenes were expressed under control of the EF-1α promoter (brown). (C) Pair-wise immunoprecipitation of comparable amounts of FLAG-tagged HDAC1/2/3 WT and CI enzymes to assess their deacetylase activity. FLAG immunoprecipitates were analyzed on Western blots (upper panel) using the FLAG antibody. Precipitates of wildtype HAP1 cells are shown as negative control. Associated deacetylase activities of HDAC WT and CI enzymes are presented below. (A+C) Bar graphs represent mean values ± standard deviation (SD) of 4 (A) or 3 (C) biological replicates and the significance was determined by Welch‘s t-test. *p < 0.05, **p < 0.01, ***p < 0.001. (TIF) [file pgen.1010376.s001.tif]

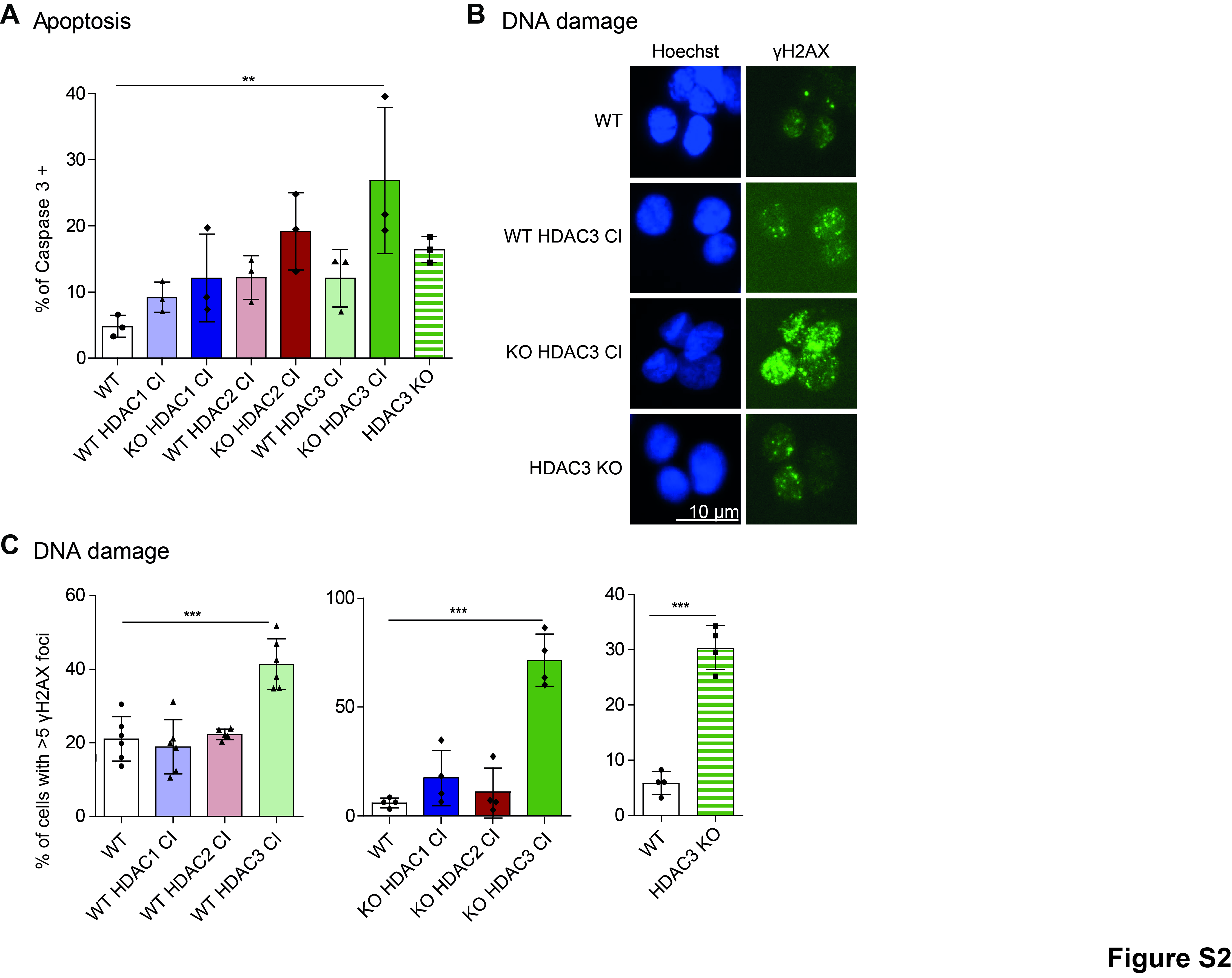

Supplement: S2 Fig — (A) Bar graph representing the percentage of apoptotic cells of cultured cell lines, including wildtype cells, HDAC1/2/3 CI expressing cells (wildtype and knockout background) and HDAC3 KO cells. Apoptotic cells were determined by flow cytometry based on signals for cleaved caspase 3. (B) Representative immunofluorescence images of wildtype control cells and cells with inactivated/deleted HDAC3. DNA damage was analyzed with an antibody specific for γ-H2AX and DNA was stained with Hoechst dye. (C) Quantification of γ-H2AX foci determined by immunofluorescence analysis from WT HDAC1/2/3 CI cells (left panel), KO HDAC1/2/3 CI cells (middle panel) and HDAC3 KO cells (right panel) next to wildtype cells as control. Mean values ±SD of 3 (A) or 4–6 (C) biological replicates are shown. Significance was determined by one-way ANOVA. The pairwise comparison of HDAC3 KO cells to wildtype cells (in (C), right panel) was performed using Welch‘s t-test. **p < 0.01, ***p < 0.001. (TIF) [file pgen.1010376.s002.tif]

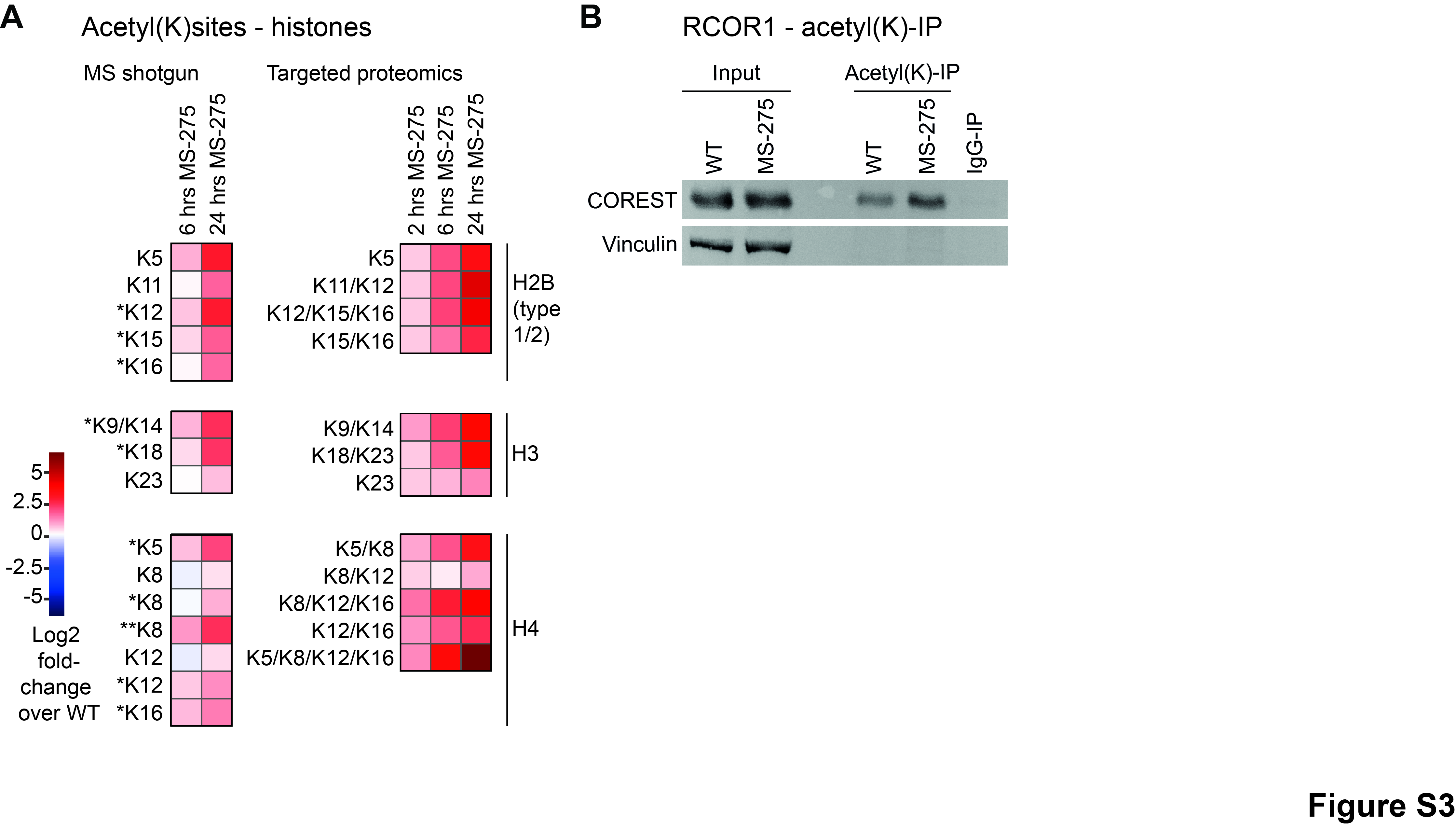

Supplement: S3 Fig — (A) Dynamics of histone acetylation. A selected set of histone acetyl(K) sites, based on findings from the MS-shotgun experiment (left panel), was further investigated by targeted proteomics to monitor changes after 2, 6 and 24 hours of MS-275 treatment (right panel, n = 4). Increased acetylation is shown as log2 fold change over untreated wildtype cells. Individual hits from histone sub-variants are summarized according to their main types (indicated on the right). Positions of acetyl-lysines in the histone protein are indicated on the left. N-terminal methionines are not considered, to fit the commonly used histone site code. *, **: The information for quantification of this site comes from a *dual or ** triple acetylated peptide where only one site was confidentially allocated (for MS shotgun data only). (B) Assessment of COREST (RCOR1) acetylation by acetyl(K)-IP and subsequent Western blot analysis using untreated or 24 hour MS-275 treated cells. The blot was incubated with indicated antibodies (on the left). Vinculin was used as loading control. n = 2 (TIF) [file pgen.1010376.s003.tif]

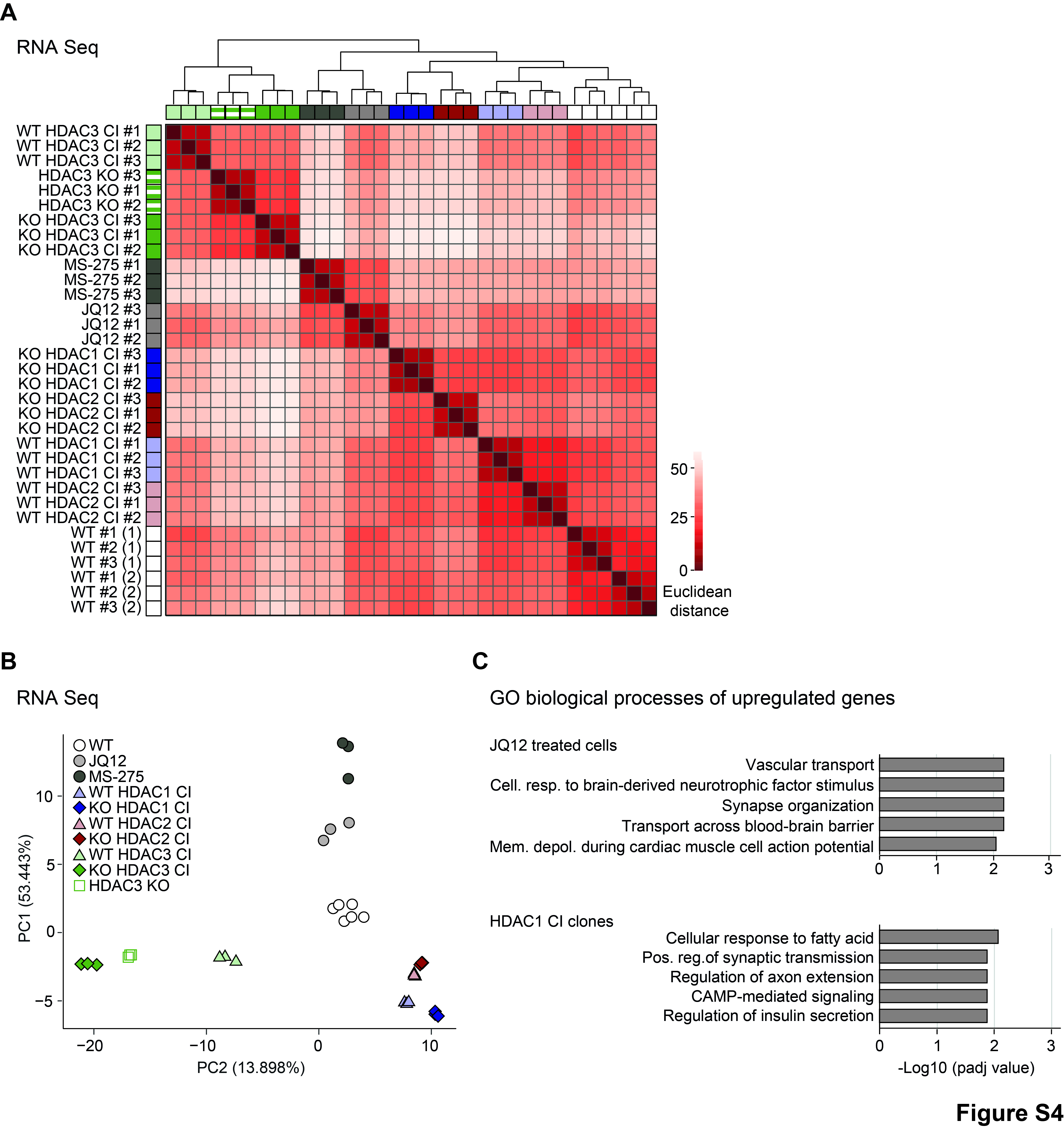

Supplement: S4 Fig — (A) Sample distance heatmap of RNA seq replicas from HDAC1/2/3 CI expressing cell lines (wildtype and knockout background), MS-275 or JQ12 treated cells (24 hours) and wildtype cells. The color code is based on the Euclidean distance of read counts after variance stabilizing transformation, while the dendrogram represents a hierarchical cluster analysis with the complete linkage method. (B) Two-dimensional principal component analysis (PCA) of RNA-seq transcriptome profiles of wildtype, MS-275 or JQ12 treated cells, HDAC1/2/3 CI expressing cells (wildtype and knockout background) and HDAC3 KO cells. (C) Enriched gene ontology terms of significantly upregulated genes by JQ12 treatment (upper panel) and HDAC1 inactivation (lower panel) (≥ 2-fold change over wildtype cells, padj-value ≤ 0.05), determined with the Enrichr tool. Only genes elevated in both HDAC1 CI clones (wildtype and knockout background) were considered for analysis. (TIF) [file pgen.1010376.s004.tif]

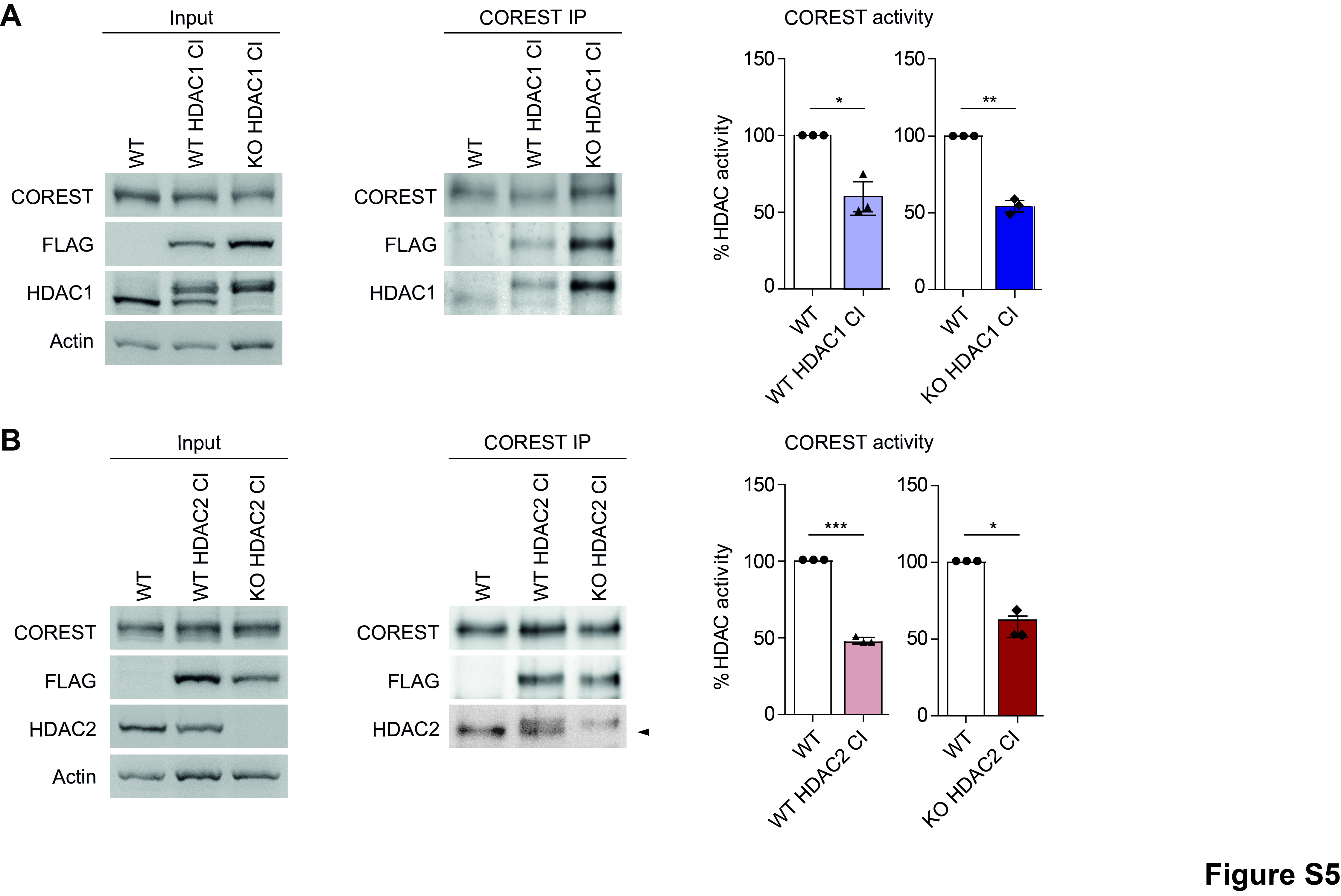

Supplement: S5 Fig — (A-B) Analysis of COREST (RCOR1) associated HDAC activity upon incorporation of inactive HDAC1 or HDAC2 enzymes. COREST was immunoprecipitated from extracts of (A) wildtype, WT HDAC1 CI and KO HDAC1 CI cells and of (B) wildtype, WT HDAC2 CI and KO HDAC2 CI cells. Inputs (left panels) and immunoprecipitates (middle panels) were analyzed by Western blotting using antibodies indicated on the left. Bar graphs (right panels) represent changes of COREST associated deacetylase activity, measured by incubating the precipitates with acetylated histones. Mean values ± SD of 3 biological replicates are shown and significance was determined by Welch‘s t-test. *p < 0.05, **p < 0.01, ***p < 0.001. (TIF) [file pgen.1010376.s005.tif]
