# Supplementary material for: A toolbox for class I HDACs reveals isoform specific roles in gene regulation and protein acetylation
Source: PLoS Genet. 2022 Aug 22;18(8):e1010376. doi: 10.1371/journal.pgen.1010376 (PMC9436093; doi:10.1371/journal.pgen.1010376)
Supplement: S1 Material — (ZIP) [file pgen.1010376.s009.zip › Supplementary_Materials_1/r_scripts_proteomics/output_html/acetylKSites_analysis_prot_norm.html]

Quantitative analysis of lysine acetlation sites in differenct HDAC mutants in HAP1 cells


# Quantitative analysis of lysine acetlation sites in differenct HDAC mutants in HAP1 cells

### submitted by Lena Hess (Seiser Lab)

#### performed by Markus Hartl - Max Perutz Labs Mass Spectrometry Facility

#### May 10, 2022

Note: This analysis was performed as an R Markdown Notebook in RStudio. Not all code will be displayed in the final report but is available as .rmd file. Parts of the code were kindly provided by Moritz Madern and further adapted.

## 1. Introduction

The experiments consists of 45 samples (15 conditions or genotypes in three biological replicates), measured as three TMT-16plex batches. The sixteenth channel was a pool of all 45 samples which was added to each 15-plex set and used for internal-reference-standard normalisation (IRS). The labeled peptides were neutral-pH reversed phase fractionated (10 pooled fractions), and enriched for acetylated peptides. This results in a total of 60 measurements ([10 fraction proteome + 10 fractions acetylome] x 3 reps). All runs were searched in MaxQuant and the result files (proteinGroups.txt, acetylKsites.txt) will be corrected for isotopic impurities during the analysis. This script/analysis deals with the acetylation sites. We follow the following strategy: 1. correction of isotopic impurity and basic filtering and data preparation, 2. Within-set median normalisation, 3. Between-set IRS normalisation, 4. Site-to-protein normalisation, 5. Differential abundance analysis using LIMMA, 6. Plotting.

## 2. Load data and correction of isotopic impurities

The data were loaded and rearranged for further processing. This includes splitting the quantitative values for singly, doubly, or multiply acetylated sites in separate entries (rows). Then, we compare the signal strength of all channels before and after impurity correction (in the same manner as for the proteome data). The data looks as expected.

## 3. Within-set median normalisation

Before normalisation entries with no quantitative information are removed (many of them were generated in the previous step were quantitative information for different acetylation states was split).Then the data were median normalized (channel-wise). In contrast to the proteome level data we do expect differences between channels in total intensities due to stronger changes in the acetylome. For example the 24h inhibitor treatment clearly showes increased intensity levels. Nevertheless, overall acetylome levels are rather stable and median normalisation is suitable (which was also inspected by scatter plots, not shown here):

### Principal component analysis

As quality control we perform PCA. As expected, we see the same TMT-labeling set dependent batch effect as on the proteome level, which makes IRS normalisation necessary. PC3 indicates there might be an additional but much weaker experimental batch effect, that we might need to deal with after IRS normalisation.

## 4. Between-set IRS normalisation

For IRS normalisation the geometric mean of all reference channel intensities is calculated (per protein), and then all values for each set are scaled to this common reference. We again inspect the results using PCA. Plotting PC1 vs. PC2 clearly shows that the TMT-set dependent batch effect was removed by the normalisation. PC3 still seems to hint at another experimental batch effect that needs to be dealt with during differential abundance analysis.

In addition we inspect the Pearson correlation between samples after normalisation. Again we observe a slight batch effect (replicate 1; compare PC3 in PCA) that needs to be considered in the linear model for differential analysis. In addition two samples appear to be in general more variable (HDC1m\_KO\_r1 and HDC1m\_KO\_r2). However, the overall higher variation at the level of lysine acetylation as compared to the proteome is expected, due to the general lower signal of lysine acetylated peptides and the additional aggregation of peptides on protein level. The clustering of experimental groups in the PCA and the overall rather high correlation indicate a reproducible dataset that allows further inspection in differential analysis.

## 5. Site-to-protein normalisation

To be able to differentiate site-level from protein level-changes, the site data are normalized to protein data. Before this step, the the acetyl-K sites are further filtered to increase reliability and robustness of the data. This means that addition to the 1% FDR cut-off and 40 score cut-off already applied in MaxQuant, we apply a score filter of >=75. An additional intensity filter is not needed as due to the combination of three datasets only sites robustly identified in all three experiments are included, which exhibit sufficient signal to noise.  
The normalised site-data are then again inspected by PCA. Similar to before we see two rather similar components (PC2 and PC3), where PC2 seems to represent the experimental batch effect observed before, and PC3 represents the sample groups. The experimental batch effect will thus be considered in the model for the LIMMA-analysis.

## 6. Differential abundance analysis using LIMMA

LIMMA is performed with the “trend” option and using the following model: (~ 0 + group + batch).

The following significantly regulated sites (at 5% FDR) were determined:

|  | X6H-CTRL | X24H-CTRL | HDC1m\_WT-CTRL | HDC1m\_KO-CTRL | HDC2m\_WT-CTRL | HDC2m\_KO-CTRL | HDC3m\_WT-CTRL | HDC3m\_KO-CTRL | HDC3\_KO-CTRL |
| --- | --- | --- | --- | --- | --- | --- | --- | --- | --- |
| Down | 2 | 45 | 5 | 72 | 10 | 71 | 246 | 2171 | 978 |
| NotSig | 13150 | 12663 | 13141 | 12730 | 13102 | 12561 | 12828 | 10140 | 11682 |
| Up | 35 | 479 | 41 | 385 | 75 | 555 | 113 | 876 | 527 |

The results are stored to: “acK-sites\_filtered\_proteinnormalized\_LIMMA.txt” as well as in a reduced format as “acK-sites\_filtered\_proteinnormalized\_LIMMA\_condensed.txt”.

### Volcano plots

Plots for all comparisons of interest are generated and stored, marking significant sites (at 5% FDR and >1.5-fold difference). Additional plots with Proteins of interested marked are also generated.
