# Supplementary material for: A toolbox for class I HDACs reveals isoform specific roles in gene regulation and protein acetylation
Source: PLoS Genet. 2022 Aug 22;18(8):e1010376. doi: 10.1371/journal.pgen.1010376 (PMC9436093; doi:10.1371/journal.pgen.1010376)
Supplement: S1 Material — (ZIP) [file pgen.1010376.s009.zip › Supplementary_Materials_1/r_scripts_proteomics/output_html/proteingroups_data_processing.html]

Quantitative proteomics analysis of different HDAC mutants in HAP1 cells


# Quantitative proteomics analysis of different HDAC mutants in HAP1 cells

### submitted by Lena Hess (Seiser Lab)

#### performed by Markus Hartl - Max Perutz Labs Mass Spectrometry Facility

#### Dec 16, 2020; updated & modified April 11 and July 9, 2022

Note: This analysis was performed as an R Markdown Notebook in RStudio. Not all code will be displayed in the final report but is available as .rmd file.

## 1. Introduction

The experiments consists of 45 samples (15 conditions or genotypes in three biological replicates), measured as three TMT-16plex batches. The sixteenth channel was a pool of all 45 samples which was added to each 15-plex set and used for internal-reference-standard normalisation (IRS). The labeled peptides were neutral-pH reversed phase fractionated (10 pooled fractions), and enriched for acetylated peptides. This results in a total of 60 measurements ([10 fraction proteome + 10 fractions acetylome] x 3 reps). All runs were searched in MaxQuant and the result files (proteinGroups.txt, acetylKsites.txt) will be corrected for isotopic impurities during the analysis.

## 2. Load data and quality control

The data were loaded and reverse database hits removed. As quality control, we will create an overview of the replicates (i.e. multiplex sets), their signal intensities and the overlap.

First, we compare the signal strength of all channels:

The distribution of channel intensities and of contaminants seems to be largely comparable, as expected. Contaminants were thus removed for further analysis steps, as well as proteins only identified by site (which cannot be quantified correctly anyway).

Here is an overview of the protein groups identified and remaining after filtering:

| total | -reverse | -reverse & con | -rev & con & only by site |
| --- | --- | --- | --- |
| 9987 | 9516 | 9439 | 8778 |

It is known that muliple TMT-sets overlap only partially due to stochasticiy in the MS/MS measurement. In this experiment we observe about 84% overlap on protein group level between the three replicates, which is in the expected range:

## 3.Correction for isotopic impurities

Isotopically labeled reagents used for TMT synthesis are not 100% pure. We correct for these isotopic impurities using the data provided by the manufacturer for the used lot of reagents, yielding corrected data matrices for all three sets.

## 4. Normalisation procedures

To be able to compare the sample we need to apply two normalisations:  
1. Global normalisation of all channels within one 16-plex set using median scaling. 2. Normalisation between different 16-plex sets using internal-reference-standard normalisation (IRS) according to Plubell et al. (doi: 10.1074/mcp.M116.065524).

### Median scaling

All channels of one set are scaled to the mean of all channel median intensities. Then only the intersection (overlap) of all three experiments is selected for IRS normalisation.

### IRS normalisation

For IRS normalisation the geometric mean of all reference channel intensities is calculated (per protein), and then all values for each set are scaled to this common reference.

To inspect the effect of IRS-normalisation we perform PCA analysis before and after the procedure.

The PCA shows that IRS-normalization removed the batch effects and that the replicates of each group cluster well in the PCA.

For further inspection of we also calculate Pearson correlation coefficients and plot them in a heatmap. As can be seen below the samples and replicates are highly correlated with all coefficients >0.97. Replicates HDC3m\_KO and HDC3\_KO are highly correlated to each other but seem to slightly differ from all other samples.

Results are stored as full matrix with corrected values:

```
write.table(df_prot_norm, file = "proteinGroups_corrected.txt", sep = "\t", col.names = TRUE, row.names=FALSE)
```

## 5. Group comparison and statistical analysis

For quantitative statistical analysis the normalised dataset was further filtered for protein groups having at least 2 razor or unique peptides and for all protein groups with an intensity >= the 0.5% quantile of the mean top 3 intense channels (6626 proteins groups remaining).

The resulting matrix was then used to calculate LIMMA statistics to determine statistically significant differential expression changes between sample groups.

The following comparisons were made:  
X6H-CTRL, X24H-CTRL, HDC1m\_WT-CTRL, HDC1m\_KO-CTRL, HDC2m\_WT-CTRL, HDC2m\_KO-CTRL, HDC3m\_WT-CTRL, HDC3m\_KO-CTRL, HDC3\_KO-CTRL

Significantly regulated proteins (at 5% FDR) for all comparisons:

|  | X6H-CTRL | X24H-CTRL | HDC1m\_WT-CTRL | HDC1m\_KO-CTRL | HDC2m\_WT-CTRL | HDC2m\_KO-CTRL | HDC3m\_WT-CTRL | HDC3m\_KO-CTRL | HDC3\_KO-CTRL |
| --- | --- | --- | --- | --- | --- | --- | --- | --- | --- |
| Down | 0 | 1183 | 865 | 1746 | 1157 | 1823 | 1246 | 2211 | 2054 |
| NotSig | 6623 | 4300 | 4805 | 3526 | 4588 | 3332 | 3506 | 1962 | 2122 |
| Up | 3 | 1143 | 956 | 1354 | 881 | 1471 | 1874 | 2453 | 2450 |

As a graphical overview, we also present the results as volcano plots with p-values (not corrected for multiple testing) or adj.p.values (corrected). Red indicates p-values or adjusted p-values below 0.05, blue above, respectively:

All data are finally stored in a matrix for further processing (including Protein group IDs & gene names):

```
df_prot_limma <- data.frame(df_p, tt_exp_limma)
write.table(df_prot_limma, file = "protein_groups_limma_results.txt", sep = "\t", row.names = FALSE, quote = FALSE)
```
